# Operative Versus Non-Operative Management for Pancreatic Trauma: An Umbrella Review of Systematic Reviews and Meta-Analyses

**DOI:** 10.3390/jcm15207865

**Published:** 2026-10-12

**Authors:** Soon Ki Min, Mina Lee, Youngmin Kim, Kang Kook Choi, Byungchul Yu, Gil Jae Lee, Wu Seong Kang

**Affiliations:** 1 Department of Trauma Surgery, Gachon University Gil Medical Center, Incheon 21565, Republic of Korea; minsk0611@gilhospital.com (S.K.M.); jwh@gilhospital.com (M.L.); skyofmay@gilhospital.com (Y.K.); calvin511@naver.com (K.K.C.); kane2123@gilhospital.com (B.Y.); nonajugi@gilhospital.com (G.J.L.); 2 Department of Traumatology, Gachon University College of Medicine, Incheon 21565, Republic of Korea

**Keywords:** pancreatic injury, non-operative management, pancreatic fistula, pancreatic pseudocyst, umbrella review

## Abstract

**Background/Objectives**: Pancreatic trauma is uncommon but highly morbid, and management has shifted toward non-operative management (NOM) with endoscopic and percutaneous adjuncts. Existing systematic reviews report heterogeneous findings, and no umbrella review has been published. We therefore synthesized this evidence, comparing operative management (OM) with NOM. **Methods**: MEDLINE, Embase, and the Cochrane Library were searched through 3 September 2026, following PRISMA 2020 and PRIOR (PROSPERO CRD420261495303). Eligible studies were systematic reviews comparing OM with NOM in pancreatic trauma. Two reviewers independently performed selection, extraction, AMSTAR 2, and ROBIS appraisal. Findings were synthesized narratively, separately for children and adults, with blunt injury as the primary comparison. **Results**: Six reviews were included (three pediatric, two adult, one mixed). In children, no review demonstrated a statistically significant difference in mortality (odds ratio [OR] for NOM vs. OM, 0.96; 95% CI, 0.42–2.22); NOM succeeded in 87% and was associated with more pseudocysts (OR, 3.05; 95% CI, 1.84–5.05; OR, 12.46 in grade III or higher injury), two-thirds managed without surgery, and with less fistula (OR, 0.27; 95% CI, 0.09–0.82). Adult estimates were crude and highly imprecise (mortality risk ratio for OM vs. NOM, 0.67; 95% CI, 0.04–9.91). Pediatric reviews overlapped heavily (corrected covered area, 38.3%); all six had critically low confidence and high risk of bias. **Conclusions**: The evidence does not allow the two strategies to be judged equivalent in either population. It is confounded by indication and concentrated in overlapping pediatric cohorts; the key unanswered question concerns stable patients with blunt ductal injury, stratified by ductal location.

## 1. Introduction

Injury remains a leading cause of death and long-term disability worldwide, and abdominal injury accounts for a disproportionate share of potentially preventable trauma deaths [1,2,3]. Within the abdomen, pancreatic injury is among the least common but most morbid diagnoses, reported in 0.2% to 12% of abdominal trauma [4], in approximately 3% to 5% of adult trauma admissions [5], and in fewer than 5% of children admitted after blunt abdominal trauma [6]. Reported mortality ranges from 9% to 34% [5,7] and is driven largely by associated injuries, whereas pancreas-specific morbidity, including fistula, pseudocyst, intra-abdominal abscess, and sepsis, determines the subsequent clinical course.

The Eastern Association for the Surgery of Trauma practice management guideline conditionally recommends non-operative management for grade I/II injuries and resection for grade III/IV injuries identified on computed tomography [4]. Applying this framework is difficult in practice. Because the pancreas is retroperitoneal and the clinical presentation is nonspecific, serum amylase and lipase are unreliable in the first hours after injury, and the sensitivity of computed tomography for pancreatic injury ranges from 47% to 79% and for ductal injury from 52% to 54% [4], a substantial proportion of significant ductal injuries are missed when imaging is performed early [8]. Delayed diagnosis is in turn associated with higher complication rates, including pancreatic fistula, peripancreatic collections and pseudocyst, and sepsis, which has caused death after an initially missed injury [4,9]. Over the same period, non-operative management has expanded with percutaneous catheter drainage, interventional radiology, and endoscopic retrograde cholangiopancreatography with ductal stenting, and incorporating endoscopic adjuncts appears to reduce both failure of non-operative management and pancreas-related complications [5].

Numerous systematic reviews have addressed this question, but they have used heterogeneous definitions and analytic approaches, drawn repeatedly on the same primary cohorts, and reached discordant conclusions. In this situation, a further systematic review of primary studies would add another synthesis of the same cohorts without explaining why the existing reviews disagree. An umbrella review addresses a different question: it appraises the methodological quality of the existing reviews, quantifies the overlap of their primary studies to show whether concordant findings represent independent replication, compares their definitions and analytic choices to explain discordance, and summarizes the certainty of the evidence reported across reviews [10,11]. No umbrella review on this question has been published to our knowledge. We therefore conducted an umbrella review comparing operative with non-operative management of pancreatic trauma, with the aim of establishing what the existing systematic reviews can and cannot support, separately for children and adults.

## 2. Materials and Methods

### 2.1. Literature Search

This umbrella review was conducted in accordance with the Preferred Reporting Items for Systematic Reviews and Meta-Analyses (PRISMA) 2020 guidelines [12] (Supplementary Table S1) and reported in accordance with the Preferred Reporting Items for Overviews of Reviews (PRIOR) statement [10] (Supplementary Table S2). The protocol was prospectively registered in the International Prospective Register of Systematic Reviews (PROSPERO; CRD420261495303; first submitted on 2 September 2026, before formal searching began, and registered on 3 September 2026); no protocol document was prepared beyond the registration record. MEDLINE via PubMed, Embase, and the Cochrane Library (including the Cochrane Database of Systematic Reviews and the Cochrane Central Register of Controlled Trials [CENTRAL]) were systematically searched for eligible studies published through 3 September 2026. The search strategy was developed a priori and combined controlled vocabulary (Medical Subject Headings [MeSH] and Emtree terms) with free-text keywords (Supplementary Table S3). To ensure comprehensive identification of studies, the reference lists of eligible articles and of relevant reviews were additionally screened by hand. Deviations from the registered protocol are described in Section 2.7.

### 2.2. Eligibility Criteria

Eligible studies were systematic reviews, with or without meta-analysis, of pediatric and/or adult patients with blunt or penetrating pancreatic trauma (American Association for the Surgery of Trauma [AAST] grades I–V) that compared operative management (OM), defined as surgical treatment directed at the injured pancreas, with non-operative management (NOM), defined as intentional observation and conservative support with endoscopic or percutaneous adjuncts, and that reported at least one outcome of interest. A systematic review was defined as a review that reported a search of at least one bibliographic database, predefined eligibility criteria, and a list of the included studies. Reviews addressing overlapping populations, interventions, comparators, or outcomes were all retained at the selection stage; overlap was managed during synthesis by quantifying primary-study overlap and by selecting a representative review for each pediatric outcome (Section 2.5). Narrative and scoping reviews, primary clinical studies, guidelines without a systematic search, reviews restricted to case reports or case series, and reviews of non-traumatic pancreatic disease were excluded. Only English-language publications were eligible, as prespecified in the protocol, because excluding non-English publications has rarely changed the conclusions of systematic reviews [13,14]; no date restriction was applied. Two reviewers (S.K.M. and M.A.L.) independently screened titles, abstracts, and full texts, with disagreements resolved by consensus or by a third reviewer (W.S.K.).

### 2.3. Data Extraction

Two investigators (S.K.M. and M.A.L.) independently extracted the data, and disagreements were resolved in the same manner. The extracted items were the first author, publication year, journal, country, review type, databases searched and search period, number of primary studies, patient characteristics and injury mechanism, AAST grade distribution, the definitions applied to operative and non-operative management, and all reported outcome data for the comparison between OM and NOM. Comparisons within operative management reported by the included reviews, such as resection vs. non-resection, octreotide prophylaxis, drain type, and splenic preservation, were outside the protocol-specified question and were not extracted. When reviews reported different data for the same primary study or outcome, data were extracted as reported by each review, and the discrepancies were noted and considered in the synthesis. The methods and results of each review’s assessment of publication bias and any certainty-of-evidence ratings (e.g., GRADE) were also extracted as reported; neither reporting bias nor certainty of evidence was assessed anew. All records retrieved from the database search were imported into EndNote version 2025 (Clarivate, Philadelphia, PA, USA) for deduplication, first automatically and then by manual inspection of the remaining records.

### 2.4. Methodological Quality and Risk of Bias Assessment

Methodological quality was appraised with AMSTAR 2 (A MeaSurement Tool to Assess systematic Reviews, version 2) [15], which comprises 16 items, 7 of which are critical domains (items 2, 4, 7, 9, 11, 13, and 15). Two investigators (S.K.M. and M.A.L.) applied the tool independently, with disagreements resolved as above. The nonrandomized wording of items 9 and 11 was applied because every included review synthesized nonrandomized studies. Four interpretive rules were fixed before the appraisal and applied consistently: an unjustified language or publication-type restriction failed item 4; pooling of crude, unadjusted event counts was rated inappropriate under item 11 unless confounder-adjusted estimates were also reported; a protocol neither registered nor publicly available was rated partial yes rather than yes under item 2; and item 13 was rated yes when the review discussed the likely impact of bias on its findings, whether or not risk of bias in individual studies had been formally assessed under item 9. Items 11, 12, and 15 were rated as not applicable for reviews that performed no quantitative synthesis. A response of no on a critical domain was counted as a critical flaw, and overall confidence was rated as high, moderate, low, or critically low following Shea et al. [15]; more than one critical flaw denoted critically low confidence. AMSTAR 2 was applied to a practice management guideline based on a systematic review [4] in the same manner as to the remaining reviews; because it pooled crude event counts into risk ratios, it was appraised as a meta-analysis.

Risk of bias in each review was additionally assessed with the Risk Of Bias In Systematic reviews (ROBIS) tool [16]. ROBIS comprises three phases: assessment of the relevance of each review to the umbrella review question (Phase 1); identification of concerns with the review process in four domains, namely study eligibility criteria, identification and selection of studies, data collection and study appraisal, and synthesis and findings (Phase 2); and a judgement of the overall risk of bias in the review (Phase 3). In Phase 1, a review whose population was restricted to one age group or one injury mechanism was rated as partially relevant. Signalling questions were answered as yes, probably yes, probably no, no, or no information, on the basis of the full report and its Supplementary Material. A domain was rated as low concern when all signalling questions were answered yes or probably yes; as high concern when any question was answered no, or probably no in a way likely to have affected which studies or results entered the review; and as unclear concern when concerns arose only from missing information. Absence of a registered or publicly available protocol was judged a high concern for the eligibility criteria domain. Language restrictions were judged under the identification and selection domain only, so that they were not counted twice. Overall risk of bias was rated as low, high, or unclear; a review was rated at low risk only when the concerns identified in Phase 2 were minor or were addressed in the interpretation of its findings.

Two investigators (S.K.M. and M.A.L.) applied AMSTAR 2 and ROBIS to every review independently, using the same prespecified rules and a structured rating form. Inter-rater agreement before consensus was quantified with unweighted Cohen’s κ [17] for the 16 AMSTAR 2 items (6 reviews × 16 items), for the 24 ROBIS signalling questions (6 reviews × 24 questions), and for the ROBIS domain-level judgements (6 reviews × 4 domains); κ was interpreted according to Landis and Koch [18], and 95% confidence intervals (CIs) were derived from the large-sample standard error. Agreement on the overall AMSTAR 2 and ROBIS ratings was expressed as the percentage of reviews rated identically, because κ is not defined when both raters assign a single category to every review. Disagreements were resolved by discussion, with a third investigator (W.S.K.) available for arbitration. Agreement statistics were calculated in R version 4.6.0 (R Foundation for Statistical Computing, Vienna, Austria).

Risk-of-bias assessments of the primary studies were extracted from each review as reported, independently by two investigators, and were not re-assessed. The tool used and the distribution of ratings were recorded for each review, and a review that did not assess, or did not report its assessment of, the risk of bias of its primary studies was recorded as such. Because all primary studies were observational and the comparisons reproduced from the reviews were unadjusted, and because estimates were taken from the reviews without re-accessing the primary studies, re-assessment was not expected to change the interpretation of the evidence.

### 2.5. Overlap Assessment

Overlap of primary studies among the included reviews was quantified using the covered area (CA) and the corrected covered area (CCA) [19]. The CCA was calculated as (N − r)/(rc − r), where N represents the total number of review-primary study entries, r the number of unique primary studies, and c the number of included reviews. Overlap was classified as slight (0–5%), moderate (6–10%), high (11–15%), or very high (>15%). Pairwise overlap between each pair of reviews was calculated as the number of shared primary studies divided by the number of distinct primary studies across both reviews. Primary-study sets were taken from each review’s list of included studies. The CCA was calculated overall and separately for the pediatric and the adult reviews; the review covering both populations contributed its pediatric and adult primary studies to the respective population-specific estimates, classified by the mean or median age reported for each study. Because the pediatric reviews overlapped substantially, one representative review was selected for each pediatric outcome: the review with the largest number of primary studies contributing to that outcome, with a pooled estimate preferred over a descriptive one and the more recent review preferred in case of a tie. Estimates from the other pediatric reviews were used only to check concordance. No selection was required for adults, because the adult reviews shared no primary study.

### 2.6. Data Synthesis

Owing to substantial clinical and methodological heterogeneity across the included reviews and to the overlap of their primary studies, quantitative re-pooling was not undertaken, as prespecified in the protocol. A de novo meta-analysis of the primary studies was also not undertaken, for four reasons: the primary studies represented the union of 6 searches of differing scope and era rather than a systematic sample of the primary literature; many did not compare OM with NOM; patient-level overlap across serial reports and national registries could not be resolved without individual patient data; and the primary studies reported almost exclusively crude, unadjusted event counts, so re-pooling would reproduce confounding by indication. Findings were synthesized narratively and structured around the primary outcomes of in-hospital mortality and post-traumatic pancreatic fistula and the additional outcomes of failure of NOM, pseudocyst and intra-abdominal abscess, length of stay (LOS), reintervention, and other complications, stratified by AAST injury grade (low grade I–II vs. high grade III–V) and by age group (adult vs. pediatric) where reported. Findings were presented separately for children and adults, with blunt injury as the primary comparison. Although our target comparison was pancreas-directed OM versus NOM in patients without an independent indication for laparotomy, some reviews used a pancreas-specific definition of NOM; for example, Koh et al. [6] classified laparotomy for associated non-pancreatic injuries as NOM when no pancreatic procedure was performed. Penetrating injury was described separately, because the pancreatic injury is then usually identified at a laparotomy performed for instability, peritonitis, or associated injuries, and non-operative management of the pancreas is rarely a comparable option. Estimates were reproduced in the direction reported by the source review, and the direction of every estimate is stated explicitly (for example, OR for NOM vs. OM); mean differences are expressed as NOM − OM. Where multiple reviews reported conflicting estimates for the same outcome, evidence concordance, search timelines, and methodological rigor were compared to explain divergence.

### 2.7. Deviations from the Protocol

The following deviations from the registered protocol were made. First, the Cochrane Library, including the Cochrane Database of Systematic Reviews, was searched rather than CENTRAL alone, because the eligible study design was the systematic review. Second, in response to peer review, findings were synthesized separately for children and adults, with blunt injury as the primary comparison and penetrating injury described separately; a representative review was selected for each pediatric outcome (Section 2.5); and the risk of bias of the included reviews was additionally assessed with ROBIS (Section 2.4). Comparisons within operative management, which were not prespecified, were not synthesized.

## 3. Results

### 3.1. Study Selection

**Figure 1:**
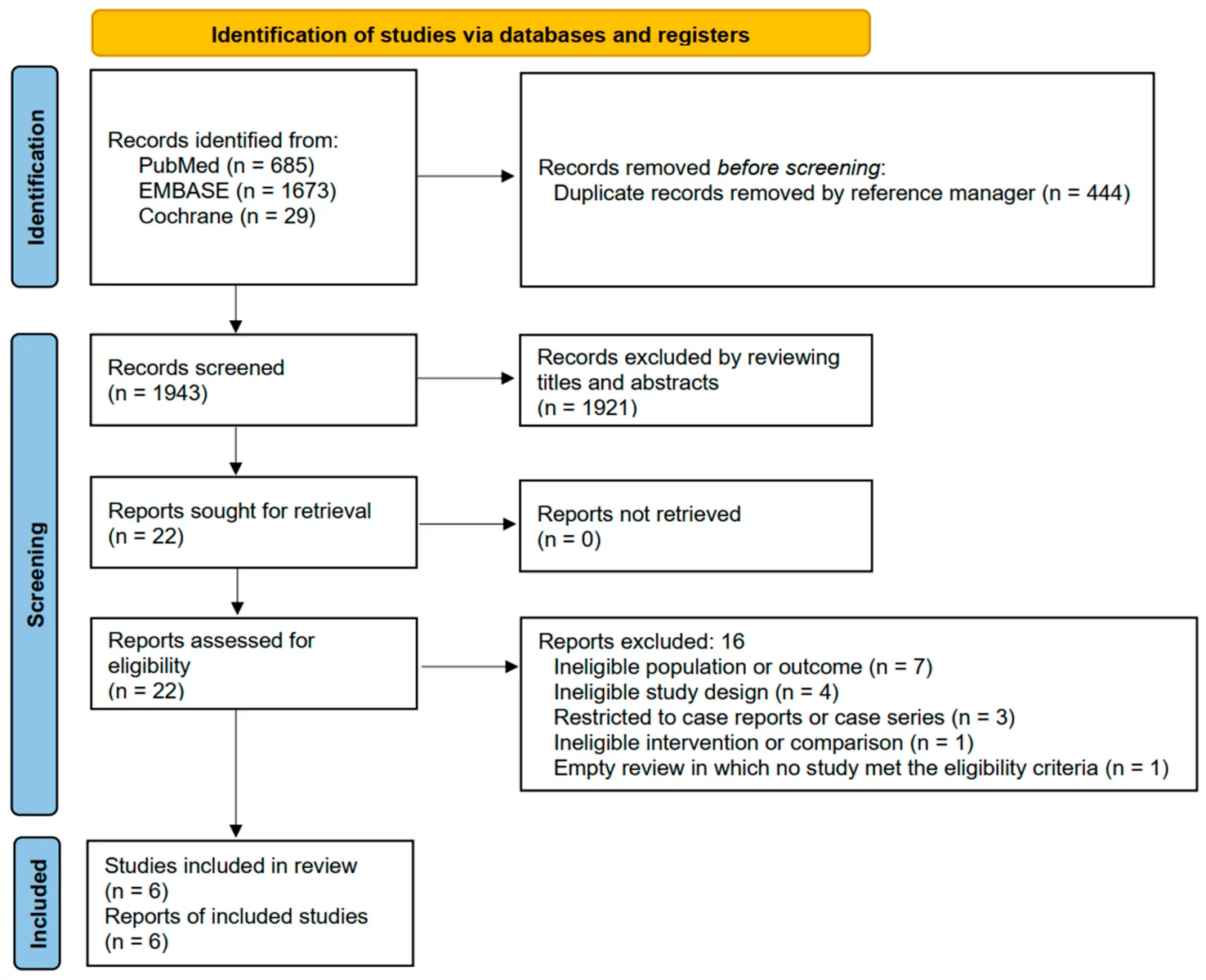
Flowchart of study selection.

After removal of 444 duplicate records, 1943 records were screened by title and abstract, and 1921 were excluded. Of the 22 reports retrieved for full-text assessment, 16 were excluded (Supplementary Table S4) [9,20,21,22,23,24,25,26,27,28,29,30,31,32,33,34]. The most common reason was an ineligible population or outcome (k = 7): 4 reviews addressed non-traumatic pancreatic disease, such as acute pancreatitis or pseudocysts of any cause [20,21,23,24], and 3 addressed other questions in trauma, namely new-onset diabetes, the staging of pancreaticoduodenectomy, and robotic surgery [28,33,34]. Four were narrative reviews, or case reports with a literature review rather than systematic reviews [25,27,29,31], 3 were restricted to case reports or case series [9,26,30], 1 addressed ERCP alone without a comparison between OM and NOM [32], and 1 was an empty review in which no study met the eligibility criteria [22]. Six systematic reviews [4,5,6,8,35,36] were included (Figure 1).

### 3.2. Characteristics of the Included Reviews

Six systematic reviews published between 2017 and 2025 were included (Table 1). Three performed a quantitative synthesis: 2 were conventional meta-analyses [8,35], and 1 pooled crude event counts into risk ratios within a GRADE-based practice management guideline [4]; the remaining 3 used narrative or descriptive synthesis [5,6,36]. The number of primary studies per review ranged from 3 to 42. Two reviews addressed adults [4,5], 3 addressed children [6,8,35], and 1 addressed both adult and pediatric patients [36]. Three reviews were restricted to blunt injury [5,6,35], 2 included both blunt and penetrating injury [4,36], and 1 placed no restriction on mechanism [8]. The operational definitions of OM and NOM differed across reviews (Supplementary Table S5). Endoscopic procedures were classified as NOM in all 6 reviews, and percutaneous procedures in the 5 that addressed them [5,6,8,35,36]. Two pediatric reviews defined OM as immediate surgery after diagnosis of pancreatic injury and counted surgery for injuries to other organs as NOM [6,35], the definition closest to the pancreas-directed definition used in this review. One adult review defined OM as laparotomy with any pancreas-related procedure [5], and 3 reviews did not define OM [4,8,36], one of which classified surgical drainage as NOM [8]. Because all reviews adopted the arm allocations reported by the primary studies, the same cohort was sometimes allocated differently; in one pediatric cohort of 79 patients, 54 were classified as non-operatively managed in one review and 47 in another [8,35]. Two reviews also reported comparisons within operative management [4,36], which were outside the review question and were not synthesized. Of the 35 primary studies in the adult guideline, 14 contributed to its comparisons of OM with NOM [4], as did 10 of the 16 in the mixed review [36]; in the other 4 reviews, all primary studies were included for this comparison [5,6,8,35].

Relative to the target population, all 6 reviews were indirect to some degree. None restricted inclusion by hemodynamic status or associated injuries, and none reported these characteristics by treatment arm. The 2 blunt-only pediatric reviews approximated the target population most closely, because they also classified surgery for injuries to other organs as NOM [6,35]. The adult guideline compared strategies only in injury graded on computed tomography, which implies that patients were stable enough for imaging, but it did not separate blunt from penetrating injury [4]. In the adult blunt-injury review, the operative group had higher injury grades and, as its authors noted, probably more associated injuries requiring laparotomy [5]. One pediatric review placed no restriction on mechanism [8], and the mixed-population review included penetrating series [36].

Two reviews included penetrating injury [4,36], but neither provided an interpretable comparison of OM with NOM for penetrating injury. The adult guideline pooled blunt and penetrating series [4], and in the mixed-population review the only penetrating comparison was a registry analysis of resection vs. non-resection presented as NOM vs. OM [36]. Penetrating injury therefore contributed no separate estimate to this synthesis.

### 3.3. Methodological Quality and Risk of Bias (AMSTAR 2 and ROBIS)

All 6 reviews were rated as having critically low overall confidence (Table 2), with 3 critical flaws in 3 reviews [6,8,36], 4 in 1 [5], 5 in 1 [35], and 6 in 1 [4]. Two items were unmet across all reviews: none provided a list of studies excluded after full-text assessment with reasons for exclusion (item 7), and none reported the funding sources of the included studies (item 10). Five reviews applied unjustified language or publication-type restrictions (item 4), the remaining review being rated partial yes [35]. Regarding protocol development (item 2), 2 reviews were registered in PROSPERO and rated partial yes [8,36], and 4 reported no protocol. Risk of bias in individual studies (item 9) was rated no in 2 reviews [4,5] and partial yes in 4, reflecting appraisal tools that were applied but incompletely reported or not linked to the synthesis. Among the 3 reviews that combined results statistically, none met item 11, because crude, unadjusted event counts were pooled without adjustment for injury grade or illness severity [4,8,35]. These unadjusted estimates should therefore be interpreted with caution, because they may primarily reflect differences in baseline injury severity, hemodynamic status, and associated injuries between the patients selected for each strategy rather than a treatment effect. Of these 3 reviews, 2 did not adequately investigate publication bias (item 15) [4,35], and 1 performed the Egger test [8]. Two reviews did not account for risk of bias when interpreting their findings (item 13) [35,36]. The 2 reviews without a study-level risk-of-bias assessment nonetheless met item 13, because one rated the risk-of-bias domain as serious for every outcome within its GRADE assessment [4] and the other attributed its findings to the lower injury grades of the non-operative group [5]. Certainty of evidence was rated very low for every outcome assessed in 2 reviews [4,8]; 1 review applied GRADE but reported the quality of evidence as moderate to high in most primary studies rather than by outcome [36], and 3 did not rate certainty [5,6,35].

**Figure 2:**
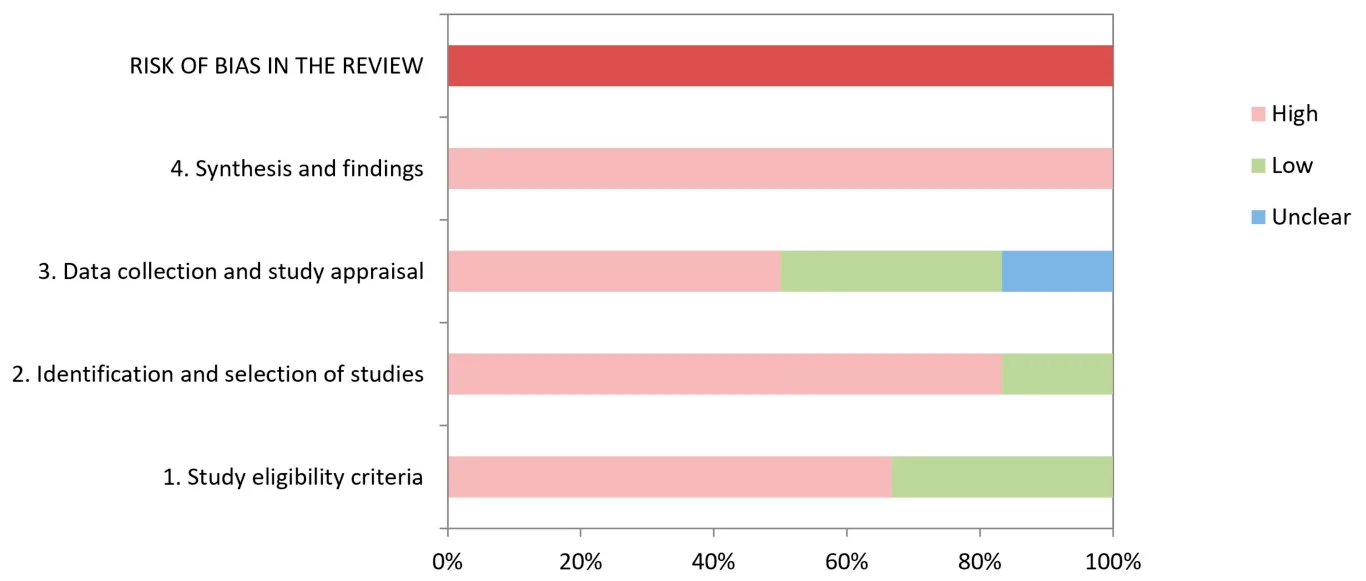
Risk of bias of the included systematic reviews assessed with ROBIS. Bars show the proportion of the 6 reviews with low, high, or unclear concern in each Phase 2 domain and with low, high, or unclear overall risk of bias (Phase 3). The top bar (dark red) shows the overall risk of bias in the review (Phase 3), which was high in all 6 reviews; the lighter colours show the level of concern in each Phase 2 domain (red, high; green, low; blue, unclear).

All 6 reviews were judged to be at high risk of bias with ROBIS (Table 3 and Figure 2), consistent with their critically low confidence on AMSTAR 2. In Phase 1, 5 reviews matched the umbrella review question only partially because they were restricted to adults [4,5] or children [6,8,35], 3 of them also to blunt injury [5,6,35], and 1 matched it fully [36].

In Phase 2, concern regarding the study eligibility criteria was high in the 4 reviews without a registered or publicly available protocol [4,5,6,35]; in 2 of these, a minimum sample size was also imposed without justification, together with a requirement for AAST grading in one [6] and for a direct comparison, applied inconsistently, in the other [5]. Concern was low in the 2 reviews registered in PROSPERO [8,36]. Concern regarding the identification and selection of studies was high in 5 reviews [4,5,6,8,36], owing to a single-database search [4], no methods additional to database searching [5,6,36], restrictive search strategies [4,5,8], unjustified language or publication-format restrictions [4,5,6,8,36], or absent or unclear independent duplicate screening [4,6,36]; one review also missed recent eligible pediatric comparative studies [8]. Only Kopljar 2021 [35], which searched several databases and reference lists without language restriction and screened in duplicate, had low concern. Concern regarding data collection and study appraisal was high in 3 reviews because the risk of bias of the primary studies was not assessed at study level [4], was listed as a criterion without reported results [5], or because too few study characteristics were reported to interpret the results [35]. It was unclear in 1 review, whose extraction and appraisal processes were not described [6], and low in 2, in which data were extracted by more than one investigator and the primary studies were appraised with an accepted tool, with efforts to minimize error [8,36]; for Hus 2025, the appraisal was reported in its Supplementary Material [36]. Concern regarding synthesis and findings was high in all 6 reviews: crude, unadjusted comparisons were pooled or summed across observational studies with different injury-grade distributions, between-study heterogeneity and the robustness of the findings were rarely examined, and no review incorporated the risk of bias of its primary studies into the synthesis.

In Phase 3, no review addressed the concerns identified in Phase 2 in its interpretation beyond acknowledging the retrospective design of the primary studies or the low certainty of the evidence, and 2 reviews emphasized statistically significant findings derived from confounded comparisons or from single retrospective studies [35,36]. The overall risk of bias was therefore rated high for every review.

All 95 primary studies were observational. Three reviews appraised them with the Newcastle-Ottawa Scale: 18 of 23 studies were rated as low quality in Koh 2017 [6] and all studies in Kopljar 2021 [35], whereas Barrientos 2025 [8] rated 8 of 17 studies as high, 8 as moderate, and 1 as low quality. Hus 2025 [36] used the Joanna Briggs Institute critical appraisal tools and reported the results only in Supplementary Material, Soon 2019 [5] listed risk-of-bias criteria without reporting the assessments, and Ho 2017 [4] rated certainty at the outcome level with GRADE without a study-level assessment.

Before consensus, the 2 investigators agreed on 80.2% of the 96 AMSTAR 2 item ratings (κ = 0.70; 95% CI, 0.58–0.82; substantial agreement) and on 59.7% of the 144 ROBIS signalling questions (κ = 0.48; 95% CI, 0.38–0.59; moderate agreement). Agreement was substantial for both the critical (κ = 0.63) and the non-critical (κ = 0.73) AMSTAR 2 items and ranged from fair to moderate across the ROBIS domains (κ = 0.33, 0.53, 0.40, and 0.58 for Domains 1 to 4, and 0.33 for Phase 3). Agreement on the ROBIS domain-level judgements was 79.2% (κ = 0.25; 95% CI, −0.33 to 0.84; 24 judgements), the low κ despite high percentage agreement reflecting the predominance of high-concern judgements. The 2 investigators assigned the same overall AMSTAR 2 rating (critically low) and the same overall ROBIS judgement (high risk of bias) to all 6 reviews. The 19 AMSTAR 2 and 64 ROBIS discrepancies were resolved by consensus without recourse to the third investigator.

### 3.4. Overlap Among Included Reviews

The 6 reviews collectively included 137 review-primary study entries, representing 95 unique primary studies (Table 4A and Table S6). The covered area was 24.0%, and the corrected covered area was 8.8%, indicating moderate overall overlap. Overlap among the pediatric evidence was very high (CCA, 38.3% among the 3 pediatric reviews, and 27.9% when the pediatric studies of the mixed-population review were included; Table 4B), whereas overlap in adults was slight (0% between the 2 adult reviews, and 1.1% when the adult studies of the mixed-population review were included; Table 4C). Pairwise overlap was very high between Koh 2017 [6] and Kopljar 2021 [35] (43.5%; 20 of 46 studies, representing 83.3% of the studies included in Koh 2017 [6]), between Kopljar 2021 [35] and Barrientos 2025 [8] (37.2%; 16 of 43, representing 94.1% of Barrientos 2025 [8]), between Koh 2017 [6] and Barrientos 2025 [8] (28.1%; 9 of 32, representing 52.9% of Barrientos 2025 [8]), and between Barrientos 2025 [8] and Hus 2025 [36] (17.9%; 5 of 28, representing 31.2% of Hus 2025 [36]). Overlap was high between Koh 2017 [6] and Hus 2025 [36] (11.1%) and moderate between Kopljar 2021 [35] and Hus 2025 [36] (9.4%). Ho 2017 [4] shared no primary study with any other included review, and Soon 2019 [5] shared a single study with Hus 2025 [36] (5.6%), reflecting the separation between the adult and pediatric literatures. Sixteen of the 17 studies in Barrientos 2025 were contained in Kopljar 2021, the only additional study being Everson 2023, and both meta-analyses reported the same fistula OR [8,35]; their concordant estimates therefore represent the same data analyzed twice rather than independent replication. Barrientos 2025 also did not include 2 pediatric database studies published in 2024 that were captured by the mixed-population review [36]. The representative review selected for each pediatric outcome is shown in Supplementary Table S7. In addition, several primary studies were likely to share patients, including serial reports from the same centers and national registry analyses that may overlap with each other and with single-center reports; because individual patient data were unavailable, this patient-level overlap could not be quantified and is not captured by the CCA.

### 3.5. In-Hospital Mortality

No included review demonstrated a statistically significant difference in mortality between strategies, but all estimates were crude, and those in adults were highly imprecise. In children, the representative meta-analysis found no difference in pancreas-related mortality (OR for NOM vs. OM, 0.96; 95% CI, 0.42–2.22) [35]; the overlapping meta-analysis and the narrative review reported concordant findings [8,36], but because 16 of the 17 primary studies of the former were shared, this concordance is not independent confirmation. In adults, no deaths occurred in either arm for grade I/II injury, and for grade III/IV injury, with 1 death in each arm, the estimate was compatible with both substantial benefit and substantial harm (RR for OM vs. NOM, 0.67; 95% CI, 0.04–9.91) [4]. Descriptive pooling in adults with blunt injury gave higher mortality after OM than after NOM (28.8% vs. 0.5%), but the non-operative group had much lower injury grades, so these figures reflect case mix rather than a treatment effect [5].

### 3.6. Pancreatic Fistula

Fistula was defined differently across reviews, so estimates are not directly comparable between children and adults. No review applied ISGPS criteria [37], and the adult guideline counted pseudocysts and peripancreatic fluid collections requiring intervention as fistula or leak [4], whereas the pediatric reviews reported pseudocysts separately [6,8,35]. In children, pancreatic fistula was less frequent after NOM in the overall analysis (OR for NOM vs. OM, 0.27; 95% CI, 0.09–0.82) [35], with a concordant estimate from the substantially overlapping meta-analysis [8]. However, when the analysis was restricted to grade III or higher injuries, no statistically significant difference was detected (OR, 0.56; 95% CI, 0.11–2.91) [35]. In adults, fistula or leak occurred in 3 of 44 patients after NOM and in none of 22 after OM with grade I/II injury, and the difference in grade III/IV injury was not statistically significant (RR for OM vs. NOM, 0.49; 95% CI, 0.23–1.03) [4].

### 3.7. Failure of Non-Operative Management

NOM succeeded in most children, and failure was consistently associated with higher injury grade. Failure was defined inconsistently: where defined, it meant surgery after an initial non-operative strategy, so percutaneous or endoscopic interventions were counted as success [6,8]; one review did not state a definition [35]; and the adult guideline did not analyze failure as a formal outcome, noting only that it occurs regularly and carries added morbidity [4]. In children, initial NOM succeeded in 87% [35], with a concordant rate in the overlapping meta-analysis [8]. Success was 96% in grade I/II and 89% in grade III–V injury, although NOM was chosen initially for only 46% of children with high-grade injury [6], and an AAST grade of III or higher was associated with failure [36].

### 3.8. Pseudocyst and Intra-Abdominal Abscess

Pseudocyst was the most consistently reported complication and was more frequent after NOM in every review, an association driven largely by high-grade injury. In children, the odds of pseudocyst were higher after NOM overall (OR for NOM vs. OM, 3.05; 95% CI, 1.84–5.05) and approximately 12 times higher in grade III or higher injury (OR, 12.46; 95% CI, 4.60–33.77) [35], with a concordant estimate from the overlapping meta-analysis [8]. The absolute difference was small in low-grade injury (9% vs. 2%) and large in high-grade injury (32% vs. 4%), and two-thirds of pseudocysts were managed without surgery [6]. In adults with blunt injury, pseudocysts were also more frequent after NOM in the 2 primary studies reported by the mixed-population review (37.5% vs. 5.2%, and 80% vs. 12.5% in grade III/IV injury) [36]. Intra-abdominal abscess was reported for the comparison in only one pediatric study of high-grade injury (8.7% after NOM vs. 0% after OM; not significant) [36].

### 3.9. Length of Stay

Length of stay did not differ overall in children but was longer after NOM in high-grade injury. Length of stay was reported in all 6 reviews but pooled in only 1. In children, the overall difference in length of stay was not statistically significant (MD for NOM minus OM, −1.26 days; 95% CI, −3.68 to 1.15) [35], consistent with a descriptive synthesis [6]. In grade III or higher injury, however, both hospital stay (MD, 7.57 days; 95% CI, 0.53–14.61; 2 studies) and parenteral nutrition (MD, 10.98 days; 95% CI, 5.48–16.48; 3 studies) were longer after NOM, that is, shorter after OM [35]. Admission to intensive care did not differ (OR for NOM vs. OM, 1.13; 95% CI, 0.10–13.1) [8]. In adults, hospital stay was shorter after NOM in descriptive pooling (10.7 vs. 21.7 days), reflecting the lower injury grades of that group [5], and could not be pooled in the adult guideline [4].

### 3.10. Reintervention

Reintervention did not differ significantly between strategies in children, and its definitions varied substantially across reviews. In children, the point estimate favored NOM, but the interval was compatible with both fewer and more reinterventions after NOM (OR for NOM vs. OM, 0.62; 95% CI, 0.21–1.79), with substantial heterogeneity [8]. Readmission did not differ (OR for NOM vs. OM, 1.08; 95% CI, 0.18–6.47; 2 studies) [35]. Reoperation for a surgical complication was required in 2% of children, and drainage of a pseudocyst was the most frequent secondary procedure [6]. In adults, descriptive pooling reported reintervention in 24.7% after NOM and none after OM, with fewer reinterventions when endoscopic therapy was incorporated into NOM [5].

### 3.11. Other Complications

Overall complications did not differ between strategies, and estimates for specific complications were too imprecise to be informative. In children, overall complications did not differ (OR for NOM vs. OM, 0.75; 95% CI, 0.41–1.38) [8]. The pooled estimates for post-traumatic hemorrhage, infection, and pancreatitis were each based on 3 or 4 studies with 86 to 116 patients, with 95% CIs spanning more than two orders of magnitude (Table 5); they are compatible with both a large increase and a large decrease in risk after NOM and cannot show whether either strategy results in more of these complications [8]. In adults, overall complications were similar in descriptive pooling (37.9% after NOM vs. 39.3% after OM) [5]. Findings by injury grade were inconsistent in both adults and children, with some studies reporting more complications after NOM in high-grade injury and others reporting the opposite [36]. Sepsis was infrequently reported [4].

Outcomes by AAST grade, corresponding broadly to ductal injury location, were reported by only 1 review, from 2 primary studies [36]. In adults, pancreas-related complications were less frequent after NOM than after resection for both grade III injury (distal ductal injury; 18% vs. 41%) and grade IV/V injury (proximal or pancreatic head injury; 9% vs. 32%). In children with grade III injury (distal ductal injury), complications occurred in 10.0% after NOM and 19.0% after resection; with grade IV injury (proximal ductal injury), they occurred in 10.2% after NOM, 21.1% after resection, and 37.5% after operative drainage. These comparisons were unadjusted.

## 4. Discussion

### 4.1. Key Findings

In this umbrella review of 6 systematic reviews encompassing 95 unique primary studies [4,5,6,8,35,36], no review demonstrated a mortality difference between non-operative and operative management in children [8,35,36], and the adult estimates were too imprecise to exclude a clinically important difference in either direction [4,5]. Because these comparisons were unadjusted and confounded by indication, they do not establish that the two strategies are equivalent. This treatment-selection bias is expected to operate in a consistent direction. Non-operative management was applied preferentially to patients with lower-grade injury, fewer associated injuries, and stable physiology, which would make it appear safer than it is for outcomes such as mortality and length of stay. Conversely, the higher frequency of pseudocyst and the longer hospital stay after non-operative management in high-grade injury were observed despite this favorable selection and are therefore unlikely to be explained by it, although detection bias may inflate the former. Residual confounding would persist even in adjusted analyses, because no included review reported hemodynamic status, associated injuries, or the location of ductal injury by treatment arm.

In children, non-operative management was associated with a higher risk of pancreatic pseudocyst in every review that examined this outcome [6,8,35,36] (OR, 3.05; 95% CI, 1.84–5.05) [35], but this association was driven largely by high-grade injury. In low-grade injury, pseudocyst occurred in 9% after NOM vs. 2% after OM, a difference that was not significant [6]; in grade III or higher injury, it occurred in 32% vs. 4% [6], and the odds were approximately 12 times higher after NOM (OR, 12.46; 95% CI, 4.60–33.77) [35]. This association should be interpreted with two considerations in mind. Pseudocyst after non-operative management is in part the expected natural history of an unresected ductal injury and is subject to detection bias, because these patients undergo more serial imaging; moreover, two-thirds of pediatric pseudocysts were managed without surgery [6]. Pancreatic fistula was less frequent after non-operative management in children (OR, 0.27; 95% CI, 0.09–0.82) [35], whereas in adults the estimate for grade III/IV injury was not statistically significant (RR for OM vs. NOM, 0.49; 95% CI, 0.23–1.03) [4]. The apparent difference between populations may reflect differing outcome definitions, since the adult guideline counted pseudocysts and collections requiring intervention as fistula [4], but also different populations, selection by injury grade and physiological stability, very small adult denominators, and possible classification of pancreatic leaks after non-operative management as pseudocysts or collections in the pediatric reviews. Because pseudocysts and fistulas do not carry equivalent clinical weight, their clinical consequences are more informative. In children with grade III or higher injury, hospital stay was 7.57 days shorter after OM (95% CI, 0.53–14.61; 2 studies) and parenteral nutrition 10.98 days shorter (95% CI, 5.48–16.48; 3 studies), whereas readmission did not differ overall (OR, 1.08; 95% CI, 0.18–6.47) [35].

Initial non-operative management succeeded in 87% of children [35] and in 96% of low-grade compared with 89% of high-grade injuries [6], with an AAST grade of III or higher consistently predicting failure [6,36], whereas reintervention and overall complication rates did not differ significantly between strategies [8]. These comparisons rest entirely on retrospective data. No randomized trial of the two strategies has been published [5,22], and every primary study synthesized by the 6 reviews was observational and subject to selection by injury grade and hemodynamic status. All 6 reviews were rated as having critically low overall confidence on AMSTAR 2 (Table 2) and a high risk of bias on ROBIS (Table 3). Two reviews rated the certainty of evidence as very low for every outcome assessed [4,8].

### 4.2. Comparison with Previous Research

The EAST practice management guideline remains the reference framework for adult pancreatic trauma, conditionally recommending non-operative management for grade I/II injuries and resection for grade III/IV injuries identified on computed tomography, on evidence its authors rated as very low certainty [4]. Our findings do not contradict these recommendations but qualify them. The guideline’s recommendation of operative management for high-grade injury rests on morbidity, because its mortality estimate (RR for OM vs. NOM, 0.67; 95% CI, 0.04–9.91) was too imprecise either to support or to exclude a difference in survival [4]. The guideline also addresses adults only, and the pediatric reviews did not reproduce its conclusions [6,8,35].

The overlap among the pediatric reviews bears directly on how their agreement should be read. The 3 pediatric reviews reached consistent conclusions on mortality, pseudocyst, and fistula [6,8,35], which might suggest robust evidence. However, 20 of the 24 primary studies in one review and 16 of the 17 in another were contained in the third, and the 2 meta-analyses reported the same fistula OR. Their concordance therefore reflects repeated analysis of largely the same cohorts rather than independent replication, and any bias in those cohorts, such as selection for NOM by injury grade, acts in the same direction in every review. A further review of the same literature would be unlikely to change these estimates; only new, independent cohorts could.

A Cochrane review posed the same question in children and, after full-text assessment of 83 reports, identified no randomized clinical trial and called for multicenter trials [22]. More than a decade later, the evidence has grown in volume but not in design: the 6 reviews synthesized here rest on 95 observational primary studies, and the trials that were called for have not been conducted.

A recent meta-analysis reported a pooled clinical success rate of 89% for ERCP in traumatic pancreatic injury [32]. This does not conflict with our findings, because it addresses how ductal injury is treated within non-operative management rather than whether non-operative management should be chosen. It does, however, bear on how current the included evidence is. Three of the 6 reviews completed their searches between 2014 and 2017 [4,5,6], and the primary studies of the adult guideline date back to the early 1970s [4], whereas only the 2 most recent reviews captured contemporary cohorts [8,36], and only 1 restricted inclusion to studies published from 2007 onward [8]. As endoscopic stenting and percutaneous drainage have become more widely used, the older reviews may not reflect the current performance of non-operative management, particularly in high-grade ductal injury.

### 4.3. Clinical Implications of Present Study

No included review demonstrated a mortality difference between strategies, but all mortality estimates were crude, confounded by indication, and, in adults, highly imprecise, so they cannot be used to reassure patients and families that survival is unaffected by the choice. Discussion should therefore focus on the morbidity reported for each strategy, while acknowledging this uncertainty. Non-operative management is a reasonable default for low-grade injury, in which it succeeded in 96% of children [6]. In high-grade injury, it still succeeded in most patients, but the risk of pseudocyst rose steeply [35], so a trial of non-operative management there presupposes the capacity to drain a pseudocyst percutaneously or endoscopically and to recognize failure early, which an AAST grade of III or higher consistently predicted [6,36]. Where those resources are unavailable, a lower threshold for early operative management of ductal injury is defensible [8]. The choice of operative procedure once operation is chosen lies outside the question addressed by this review.

### 4.4. Future Research Directions

The boundary of non-operative management is likely to move as diagnosis and minimally invasive therapy improve. Non-invasive assessment of ductal integrity with MRCP, percutaneous catheter drainage of post-traumatic collections, and continued refinement of ERCP technique and stent design should together widen the range of injuries that can be managed without operation. Evaluating these developments will require prospective comparative data rather than further synthesis of retrospective cohorts. Randomized trials remain difficult, given the rarity of pancreatic injury, the acuity of presentation, and the difficulty of maintaining equipoise in high-grade injury. Prospective multicenter registries with standardized outcome definitions and adjustment for injury severity are the more realistic route to firm evidence.

### 4.5. Limitations

Several limitations should be considered. First, the search was limited to 3 databases and to English-language publications, and grey literature and registers of ongoing reviews were not searched. The language restriction was prespecified and was adopted because excluding non-English publications has rarely changed the conclusions of systematic reviews [13,14], and a justified restriction is permitted under AMSTAR 2 item 4; nonetheless, eligible reviews published in other languages or indexed only in other databases may have been missed. Second, every comparative estimate reproduced here was crude and unadjusted for injury grade, hemodynamic status, or associated injuries. Because non-operative management was preferentially applied to lower-grade injury, the large crude differences reported in adults reflect selection rather than a treatment effect, and such estimates should not be used for individual prognostication or for quantifying the risk attributable to either strategy. Moreover, in-hospital mortality, one of the two primary outcomes, has limited construct validity as a measure of pancreatic management, because deaths after pancreatic trauma are driven largely by associated injuries [5,7]; only one review restricted mortality to pancreas-related deaths [35], and another included pancreas-related or unspecified deaths [4]. The absence of a mortality difference therefore cannot be interpreted as equivalence. Third, the included reviews applied heterogeneous definitions of both exposure and outcome: no review used ISGPS criteria [37] for pancreatic fistula, none standardized the diagnosis of pseudocyst, and the operational definitions of OM and NOM differed (Supplementary Table S5), so outcomes bearing the same name are not strictly comparable across reviews. Pseudocyst is also subject to detection bias, because non-operatively managed patients undergo more serial imaging, and the drain-amylase criterion of the ISGPS definition cannot be applied to patients managed without an operative drain, so fistula cannot be ascertained symmetrically in the two arms. Fourth, overlap of primary studies was concentrated among the pediatric reviews (CCA, 38.3%), so their concordant estimates represent re-analyses of shared cohorts rather than independent replication. We addressed this by selecting one representative review for each pediatric outcome, a rule that was not prespecified. In addition, several primary studies were serial reports from the same centers or analyses of potentially overlapping national registries, so patient-level overlap, which the CCA does not capture, may have led to double counting within individual reviews. Fifth, most reviews grouped grades III to V, or III and IV, as high-grade injury, combining distal ductal injury, for which distal pancreatectomy is straightforward and endoscopic stenting often feasible, with proximal and ampullary injury, for which resection means pancreaticoduodenectomy. The location of the ductal disruption, rather than grade alone, drives the choice of strategy, yet only 2 primary studies within 1 review reported outcomes by ductal location [36]. Sixth, the primary studies spanned approximately 6 decades [4], during which computed tomography, MRCP, endoscopic stenting, and percutaneous drainage changed substantially, so older estimates may understate the current performance of NOM. Only 1 review restricted inclusion to the modern era [8], and its concordance with an unrestricted review [35] cannot be taken as evidence against an era effect, because 16 of its 17 primary studies were shared. Seventh, primary studies were not re-accessed, and their risk of bias was taken from the reviews rather than re-assessed, so primary studies that a review did not appraise remain unappraised; certainty of evidence was likewise taken from the reviews rather than rated anew, and all estimates are therefore reproduced as reported and are subject to any errors within the source reviews. Finally, AMSTAR 2 and ROBIS appraise the conduct of the reviews rather than the validity of the primary-study results, and quantitative re-pooling was not undertaken (Section 2.6), so the narrative approach adopted here cannot resolve discordance between reviews.

## 5. Conclusions

In this umbrella review comparing operative with non-operative management of pancreatic trauma, no review demonstrated a mortality difference in children, and adult estimates were too imprecise to be informative; the evidence does not allow the two strategies to be judged equivalent in either population. In children, non-operative management succeeded in most patients and was associated with more pancreatic pseudocysts, particularly in high-grade injury, most of which were managed without surgery, and with fewer pancreatic fistulas as defined by the included reviews. The available evidence is confounded by indication and concentrated in overlapping pediatric cohorts, and it rests on retrospective, unadjusted comparisons synthesized in reviews of critically low methodological confidence, with heterogeneous definitions of exposure and outcome. Prospective studies of hemodynamically stable patients with blunt pancreatic ductal injury and no other indication for laparotomy, stratified by ductal location, using standardized outcome definitions and adjustment for injury grade, are needed to define which patients benefit from each strategy.

## Figures and Tables

**Table 1:** Characteristics of the included systematic reviews.

No.	First Author (Year)	Country	Review Type	Databases; Search Period	Studies (*n*)	Population; Injury	Comparison (Definition of NOM)	Main Findings (Effect Size, 95% CI)	AMSTAR-2
1	Ho (2017) [4]	USA	SR with pooled crude risk ratios; EAST guideline (GRADE)	MEDLINE (PubMed); Jan 1965 to Dec 2014	35 (14) ^a^	Adults; blunt and penetrating; AAST grade I–V	OM vs. NOM for CT-diagnosed injury (PICO 1–2); OM and NOM not defined	Grade I/II: no deaths; NOM conditionally recommended. Grade III/IV, OM vs. NOM: death RR 0.67 (0.04–9.91); fistula RR 0.49 (0.23–1.03); OM conditionally recommended. Certainty very low.	Critically low
2	Koh (2017) [6]	The Netherlands	SR	PubMed, Embase, Cochrane Library; inception to Sep 2016	24	Children; blunt; AAST grade I–V	Immediate OM vs. NOM (observation, endoscopic or IR procedures, surgery for other injuries)	NOM success 96% (grade I/II) and 89% (grade III–V). Pseudocyst 18% vs. 4%, NOM vs. OM; two-thirds managed without surgery. No meta-analysis.	Critically low
3	Soon (2019) [5]	Australia	SR	MEDLINE, Embase, Cochrane; to Jan 2017	3	Adults; blunt; AAST grade I–V	OM (laparotomy and any pancreas-related procedure) vs. NOM (supportive care, radiological or endoscopic drainage)	Descriptive pooling only; NOM group had lower injury grades. Mortality 28.8% vs. 0.5%, OM vs. NOM. No meta-analysis.	Critically low
4	Kopljar (2021) [35]	Croatia	SR + MA	PubMed, Scopus, CINAHL, Cochrane Library; 1990 to Feb 2019	42	Children <18 years; blunt	Initial OM (immediate surgery) vs. NOM (observation, medical, endoscopic or IR treatment, surgery for other organs)	NOM vs. OM: pancreas-related mortality OR 0.96 (0.42–2.22); pseudocyst OR 3.05 (1.84–5.05), grade ≥III 12.46 (4.60–33.77); fistula OR 0.27 (0.09–0.82). NOM success 87%.	Critically low
5	Barrientos (2025) [8]	Colombia	SR + MA	MEDLINE, Embase, Scopus, CENTRAL, LILACS, Web of Science, ClinicalTrials.gov; 2007 to Sep 2025	17	Children ≤18 years; any mechanism	NOM (medical therapy, ERCP or stent, percutaneous or surgical drainage, supportive care) vs. OM (not defined)	NOM vs. OM: mortality OR 0.89 (0.48–1.66); pseudocyst OR 2.56 (1.55–4.23); fistula OR 0.27 (0.11–0.69); reintervention OR 0.62 (0.21–1.79). Certainty very low.	Critically low
6	Hus (2025) [36]	USA	SR	PubMed, Embase, Cochrane, ProQuest, Google Scholar; inception to May 2025	16 (10) ^a^	Adults and children; blunt and penetrating	NOM (conservative, ERCP, percutaneous drainage) vs. OM (not defined)	Narrative synthesis; no meta-analysis. Pseudocyst more frequent after NOM in adult blunt injury (37.5% vs. 5.2%) and pediatric high-grade injury (44% vs. 0%).	Critically low

Abbreviations: AAST, American Association for the Surgery of Trauma; AMSTAR-2, A MeaSurement Tool to Assess systematic Reviews, version 2; CENTRAL, Cochrane Central Register of Controlled Trials; CI, confidence interval; CT, computed tomography; EAST, Eastern Association for the Surgery of Trauma; ERCP, endoscopic retrograde cholangiopancreatography; GRADE, Grading of Recommendations Assessment, Development and Evaluation; IR, interventional radiology; LILACS, Latin American and Caribbean Health Sciences Literature; MA, meta-analysis; NOM, non-operative management; OM, operative management; OR, odds ratio; PICO, population, intervention, comparator, outcome; RR, risk ratio; SR, systematic review. ^a^ Number of primary studies contributing to the comparison of OM with NOM. Ratios are expressed for the first-named strategy relative to the second (e.g., OR for NOM vs. OM < 1 indicates lower odds after NOM). Mean differences are NOM − OM.

**Table 2:** Methodological quality of the included systematic reviews (AMSTAR-2).

First Author (Year)	1	2 *	3	4 *	5	6	7 *	8	9 *	10	11 *	12	13 *	14	15 *	16	Overall Confidence
Ho (2017) [4]	Y	N	N	N	N	Y	N	N	N	N	N	N	Y	N	N	Y	Critically low
Koh (2017) [6]	Y	N	N	N	N	N	N	N	P	N	NM	NM	Y	N	NM	Y	Critically low
Soon (2019) [5]	Y	N	Y	N	Y	N	N	P	N	N	NM	NM	Y	Y	NM	Y	Critically low
Kopljar (2021) [35]	Y	N	N	P	Y	N	N	N	P	N	N	N	N	N	N	Y	Critically low
Barrientos (2025) [8]	Y	P	Y	N	Y	Y	N	P	P	N	N	N	Y	Y	Y	Y	Critically low
Hus (2025) [36]	Y	P	N	N	N	N	N	P	P	N	NM	NM	N	Y	NM	Y	Critically low
Summary																	High 0, Moderate 0, Low 0, Critically low 6

Abbreviations: N, no; NM, no meta-analysis conducted; P, partial yes; Y, yes. An asterisk (*) marks the seven critical domains. Overall confidence: high, no or one non-critical weakness; moderate, more than one non-critical weakness; low, one critical flaw with or without non-critical weaknesses; critically low, more than one critical flaw. A partial yes on a critical item was counted as a weakness, not a flaw. AMSTAR-2 items: 1, PICO components in the research question and inclusion criteria. 2 *, Protocol established before the review, with significant deviations justified. 3, Explanation for the selection of study designs. 4 *, Comprehensive literature search strategy. 5, Study selection performed in duplicate. 6, Data extraction performed in duplicate. 7 *, List of excluded studies with justification. 8, Included studies described in adequate detail. 9 *, Satisfactory technique for assessing risk of bias in individual studies. 10, Sources of funding of the included studies reported. 11 *, Appropriate methods for statistical combination of results. 12, Impact of risk of bias in individual studies on the meta-analysis assessed. 13 *, Risk of bias accounted for when interpreting and discussing the results. 14, Satisfactory explanation for, and discussion of, heterogeneity. 15 *, Adequate investigation of publication bias and discussion of its impact. 16, Conflicts of interest and funding of the review reported.

**Table 3:** Risk of bias of the included systematic reviews assessed with ROBIS.

Review	Phase 1: Relevance	D1 Study Eligibility Criteria	D2 Identification and Selection of Studies	D3 Data Collection and Study Appraisal	D4 Synthesis and Findings	Phase 3: Risk of Bias in the Review
Ho (2017) [4]	Partial	High	High	High	High	High
Koh (2017) [6]	Partial	High	High	Unclear	High	High
Soon (2019) [5]	Partial	High	High	High	High	High
Kopljar (2021) [35]	Partial	High	Low	High	High	High
Barrientos (2025) [8]	Partial	Low	High	Low	High	High
Hus (2025) [36]	Yes	Low	High	Low	High	High

Domains 1–4 (Phase 2) are rated as low, high, or unclear concern; Phase 3 gives the overall risk of bias. Phase 1 indicates whether the review matched the umbrella review question (Yes) or only partially (Partial). Judgements are the consensus of 2 independent investigators.

**Table 4:** (A) Overlap of primary studies between the included systematic reviews. (B) Overlap of primary studies between the included systematic reviews: pediatric population. (C) Overlap of primary studies between the included systematic reviews: adult population.

**(A)**							
**Systematic Review**	**Primary Studies (k)**	**Ho (2017) [4]**	**Koh (2017) [6]**	**Soon (2019) [5]**	**Kopljar (2021) [35]**	**Barrientos (2025) [8]**	**Hus (2025) [36]**
Ho (2017) [4]	35	–	0.0% (0/59)	0.0% (0/38)	0.0% (0/77)	0.0% (0/52)	0.0% (0/51)
Koh (2017) [6]	24		–	0.0% (0/27)	**43.5% (20/46)**	**28.1% (9/32)**	**11.1% (4/36)**
Soon (2019) [5]	3			–	0.0% (0/45)	0.0% (0/20)	5.6% (1/18)
Kopljar (2021) [35]	42				–	**37.2% (16/43)**	9.4% (5/53)
Barrientos (2025) [8]	17					–	**17.9% (5/28)**
Hus (2025) [36]	16						–
**(B)**							
**Systematic review**	**Primary studies (k)**	**Koh (2017) [6]**	**Kopljar (2021) [35]**	**Barrientos (2025) [8]**	**Hus (2025) [36], pediatric studies**		
Koh (2017) [6]	24	–	**43.5% (20/46)**	**28.1% (9/32)**	**14.8% (4/27)**		
Kopljar (2021) [35]	42		–	**37.2% (16/43)**	**11.4% (5/44)**		
Barrientos (2025) [8]	17			–	**26.3% (5/19)**		
Hus (2025) [36], pediatric studies	7				–		
**(C)**							
**Systematic review**	**Primary studies (k)**	**Ho (2017) [4]**	**Soon (2019) [5]**	**Hus (2025) [36], adult studies**			
Ho (2017) [4]	35	–	0.0% (0/38)	0.0% (0/44)			
Soon (2019) [5]	3		–	9.1% (1/11)			
Hus (2025) [36], adult studies	9			–			

Abbreviations: c, number of included systematic reviews; CA, covered area; CCA, corrected covered area; k, number of primary studies included in the review; N, total number of review-primary study entries; r, number of unique primary studies. Values are pairwise overlap: primary studies shared by the two reviews divided by the number of distinct primary studies across both (shared/union); values above 10% are in bold. Overall: N = 137, r = 95, c = 6; CA = 24.0%; CCA = 8.8% (moderate overlap). Overlap by population is shown in Table 4B (children) and Table 4C (adults). CA = N/(r × c); CCA = (N − r)/(r × c − r). Overlap categories: 0–5%, slight; 6–10%, moderate; 11–15%, high; >15%, very high. Primary-study sets were taken from each review’s list of included studies. Abbreviations: c, number of included systematic reviews; CA, covered area; CCA, corrected covered area; k, number of primary studies included in the review; N, total number of review-primary study entries; r, number of unique primary studies. Values are pairwise overlap: primary studies shared by the two reviews divided by the number of distinct primary studies across both (shared/union); values above 10% are in bold. For pairs above 15%, the shared studies as a share of the smaller review: Koh (2017) [6] and Kopljar (2021) [35], 83.3% of Koh (2017) [6] (20/24); Kopljar (2021) [35] and Barrientos (2025) [8], 94.1% of Barrientos (2025) [8] (16/17); Koh (2017) [6] and Barrientos (2025) [8], 52.9% of Barrientos (2025) [8] (9/17); Barrientos (2025) [8] and Hus (2025) [36], pediatric studies, 71.4% of Hus (2025) [36], pediatric studies (5/7). Overall: N = 90, r = 49, c = 4; CA = 45.9%; CCA = 27.9% (very high overlap). CA = N/(r × c); CCA = (N − r)/(r × c − r). Overlap categories: 0–5%, slight; 6–10%, moderate; 11–15%, high; >15%, very high. Reviews of children: Koh (2017) [6], Kopljar (2021) [35] and Barrientos (2025) [8]. Hus (2025) [36] included adult and pediatric studies; only its 7 pediatric studies are counted here. Abbreviations: c, number of included systematic reviews; CA, covered area; CCA, corrected covered area; k, number of primary studies included in the review; N, total number of review-primary study entries; r, number of unique primary studies. Values are pairwise overlap: primary studies shared by the two reviews divided by the number of distinct primary studies across both (shared/union). Overall: N = 47, r = 46, c = 3; CA = 34.1%; CCA = 1.1% (slight overlap). CA = N/(r × c); CCA = (N − r)/(r × c − r). Overlap categories: 0–5%, slight; 6–10%, moderate; 11–15%, high; >15%, very high. Reviews of adults: Ho (2017) [4] and Soon (2019) [5]. Hus (2025) [36] included adult and pediatric studies; only its 9 adult studies are counted here.

**Table 5:** Summary of findings by outcome and population.

Population	Outcome	Estimate (Direction)	Representative Review [Concordant]	Overlap	Certainty Reported by Review	Main Limitations
Children	Mortality (pancreas-related)	OR 0.96 (0.42–2.22), NOM vs. OM	Kopljar [35] [Barrientos [8] OR 0.89 (0.48–1.66); Hus [36]]	16/17 Barrientos studies in Kopljar [35]	Not rated [Barrientos [8]: very low]	Unadjusted; pancreas-related deaths only
Children	Fistula	OR 0.27 (0.09–0.82), NOM vs. OM; grade ≥ III OR 0.56 (0.11–2.91)	Kopljar [35] [Barrientos [8] OR 0.27 (0.11–0.69)]	As above	Not rated [Barrientos [8]: very low]	Unadjusted; no ISGPS definition; asymmetric ascertainment
Children	Pseudocyst	OR 3.05 (1.84–5.05); grade ≥ III 12.46 (4.60–33.77), NOM vs. OM	Kopljar [35] [Barrientos [8] OR 2.56 (1.55–4.23); Koh [6] 18% vs. 4%]	Pediatric CCA 38.3%	Not rated [Barrientos [8]: very low]	Unadjusted; detection bias; no standard definition
Children	NOM success	87% (single-arm)	Kopljar [35] [Barrientos [8] 83.4%; Koh [6] 96% grade I/II, 89% grade III–V]	Pediatric CCA 38.3%	Not rated	No comparator; definition of failure varies
Children	Length of stay	MD (NOM − OM) −1.26 d (−3.68 to 1.15); grade ≥ III 7.57 d (0.53–14.61; 2 studies)	Kopljar [35]	–	Not rated	Unadjusted; heterogeneity; few studies
Children	TPN duration, grade ≥ III	MD (NOM − OM) 10.98 d (5.48–16.48; 3 studies)	Kopljar [35]	–	Not rated	Few studies
Children	Readmission	OR 1.08 (0.18–6.47), NOM vs. OM	Kopljar [35]	–	Not rated	Imprecision; 2 studies
Children	Reintervention	OR 0.62 (0.21–1.79), NOM vs. OM	Barrientos [8]	–	Very low	Heterogeneity; varying definitions; imprecision
Children	Overall complications	OR 0.75 (0.41–1.38), NOM vs. OM	Barrientos [8]	–	Very low	Heterogeneity; varying definitions
Children	Hemorrhage; infection; pancreatitis	OR 6.12 (0.11–337.0); 0.16 (0.003–9.09); 1.08 (0.02–49.18), NOM vs. OM	Barrientos [8]	–	Very low	Very serious imprecision (86–116 patients)
Adults	Mortality	Grade I/II: 0 deaths in either arm; grade III/IV: RR 0.67 (0.04–9.91), OM vs. NOM	Ho [4] [Soon [5]: 28.8% vs. 0.5%, descriptive]	Adult CCA 0% (1.1% incl. Hus [36])	Very low (Ho [4])	Unadjusted; mixed mechanism; 1 death per arm
Adults	Fistula or leak	Grade III/IV: RR 0.49 (0.23–1.03), OM vs. NOM	Ho [4]	As above	Very low (Ho [4])	Pseudocyst counted as fistula; imprecision
Adults	Pseudocyst	37.5% vs. 5.2%; grade III/IV 80% vs. 12.5%, NOM vs. OM (2 studies)	Hus [36]	–	Moderate to high (study level; Hus [36])	Unadjusted; 2 studies
Adults	Length of stay; reintervention	10.7 vs. 21.7 d; 24.7% vs. 0%, NOM vs. OM (descriptive)	Soon [5]	–	Not rated	Descriptive; confounded by injury grade

Abbreviations: CCA, corrected covered area; MD, mean difference; NOM, non-operative management; OM, operative management; OR, odds ratio; RR, risk ratio; TPN, total parenteral nutrition. Values in parentheses are 95% confidence intervals. Certainty is shown as rated by the source review; no overview-level rating was performed.

## Data Availability

All data analysed during this study are included in this published article and its Supplementary Information files.

## Supplementary Materials

The following supporting information can be downloaded at: https://www.mdpi.com/article/10.3390/jcm15207865/s1, Table S1: PRISMA 2020 checklist [38]; Table S2: PRIOR checklist; Table S3: Search strategy; Table S4: Systematic reviews excluded after full-text assessment, with reasons (n = 16); Table S5: Operational definitions of OM and NOM in the included reviews; Table S6: Primary studies included in the systematic reviews (n = 95); Table S7: Representative review for each pediatric outcome.

## Abbreviations

The following abbreviations are used in this manuscript:

AAST	American Association for the Surgery of Trauma
AMSTAR 2	A MeaSurement Tool to Assess systematic Reviews, version 2
CA	covered area
CCA	corrected covered area
CENTRAL	Cochrane Central Register of Controlled Trials
CI	confidence interval
CT	computed tomography
EAST	Eastern Association for the Surgery of Trauma
ERCP	endoscopic retrograde cholangiopancreatography
GRADE	Grading of Recommendations, Assessment, Development and Evaluations
ICU	intensive care unit
ISGPS	International Study Group of Pancreatic Surgery
LOS	length of stay
MD	mean difference
MeSH	Medical Subject Headings
MRCP	magnetic resonance cholangiopancreatography
NOM	non-operative management
NOS	Newcastle-Ottawa Scale
OM	operative management
OR	odds ratio
PRIOR	Preferred Reporting Items for Overviews of Reviews
PRISMA	Preferred Reporting Items for Systematic Reviews and Meta-Analyses
PROSPERO	International Prospective Register of Systematic Reviews
ROBIS	Risk Of Bias In Systematic reviews
RR	risk ratio
TPN	total parenteral nutrition
