# Supplementary material for: Operative Versus Non-Operative Management for Pancreatic Trauma: An Umbrella Review of Systematic Reviews and Meta-Analyses
Source: J Clin Med. 2026 Oct 12;15(20):7865. doi: 10.3390/jcm15207865 (PMC13705537; doi:10.3390/jcm15207865)
Supplement: Supplementary file 1 [file jcm-15-07865-s001.zip › jcm-4599740-supplementary.pdf]

Supplementary Table S1. PRISMA 2020 checklist

Section and topic	Item #	Checklist item	Location where item is reported (page)
**TITLE**			
Title	1	Identify the report as a systematic review.	1
**ABSTRACT**			
Abstract	2	See the PRISMA 2020 for Abstracts checklist.	1
**INTRODUCTION**			
Rationale	3	Describe the rationale for the review in the context of existing knowledge.	2
Objectives	4	Provide an explicit statement of the objective(s) or question(s) the review addresses.	2
**METHODS**			
Eligibility criteria	5	Specify the inclusion and exclusion criteria for the review and how studies were grouped for the syntheses.	3, 5–6
Information sources	6	Specify all databases, registers, websites, organisations, reference lists and other sources searched or consulted to identify studies. Specify the date when each source was last searched or consulted.	2–3
Search strategy	7	Present the full search strategies for all databases, registers and websites, including any filters and limits used.	3
Selection process	8	Specify the methods used to decide whether a study met the inclusion criteria of the review, including how many reviewers screened each record and each report retrieved, whether they worked independently, and if applicable, details of automation tools used in the process.	3
Data collection process	9	Specify the methods used to collect data from reports, including how many reviewers collected data from each report, whether they worked independently, any processes for obtaining or confirming data from study investigators, and if applicable, details of automation tools used in the process.	3–4
Data items	10a	List and define all outcomes for which data were sought. Specify whether all results that were compatible with each outcome domain in each study were sought (e.g. for all measures, time points, analyses), and if not, the methods used to decide which results to collect.	3, 5
	10b	List and define all other variables for which data were sought (e.g. participant and intervention characteristics, funding sources). Describe any assumptions made about any missing or unclear information.	3
Study risk of bias assessment	11	Specify the methods used to assess risk of bias in the included studies, including details of the tool(s) used, how many reviewers assessed each study and whether they worked independently, and if applicable, details of automation tools used in the process.	4–5
Effect measures	12	Specify for each outcome the effect measure(s) (e.g. risk ratio, mean difference) used in the synthesis or presentation of results.	6
Synthesis methods	13a	Describe the processes used to decide which studies were eligible for each synthesis (e.g. tabulating the study intervention characteristics and comparing against the planned groups for each synthesis (item #5)).	5–6

Section and topic	Item #	Checklist item	Location where item is reported (page)
	13b	Describe any methods required to prepare the data for presentation or synthesis, such as handling of missing summary statistics, or data conversions.	3, 6
	13c	Describe any methods used to tabulate or visually display results of individual studies and syntheses.	5
	13d	Describe any methods used to synthesize results and provide a rationale for the choice(s). If meta-analysis was performed, describe the model(s), method(s) to identify the presence and extent of statistical heterogeneity, and software package(s) used.	5
	13e	Describe any methods used to explore possible causes of heterogeneity among study results (e.g. subgroup analysis, meta-regression).	5–6
	13f	Describe any sensitivity analyses conducted to assess robustness of the synthesized results.	Not applicable
Reporting bias assessment	14	Describe any methods used to assess risk of bias due to missing results in a synthesis (arising from reporting biases).	3–4
Certainty assessment	15	Describe any methods used to assess certainty (or confidence) in the body of evidence for an outcome.	3
**RESULTS**			
Study selection	16a	Describe the results of the search and selection process, from the number of records identified in the search to the number of studies included in the review, ideally using a flow diagram.	6–7
	16b	Cite studies that might appear to meet the inclusion criteria, but which were excluded, and explain why they were excluded.	6
Study characteristics	17	Cite each included study and present its characteristics.	7–10
Risk of bias in studies	18	Present assessments of risk of bias for each included study.	11–14
Results of individual studies	19	For all outcomes, present, for each study: (a) summary statistics for each group (where appropriate) and (b) an effect estimate and its precision (e.g. confidence/credible interval), ideally using structured tables or plots.	16–19
Results of syntheses	20a	For each synthesis, briefly summarise the characteristics and risk of bias among contributing studies.	14, 18–19
	20b	Present results of all statistical syntheses conducted. If meta-analysis was done, present for each the summary estimate and its precision (e.g. confidence/credible interval) and measures of statistical heterogeneity. If comparing groups, describe the direction of the effect.	Not applicable
	20c	Present results of all investigations of possible causes of heterogeneity among study results.	Not applicable
	20d	Present results of all sensitivity analyses conducted to assess the robustness of the synthesized results.	Not applicable
Reporting biases	21	Present assessments of risk of bias due to missing results (arising from reporting biases) for each synthesis assessed.	11
Certainty of evidence	22	Present assessments of certainty (or confidence) in the body of evidence for each outcome assessed.	11, 18–19
**DISCUSSION**			
Discussion	23a	Provide a general interpretation of the results in the context of other evidence.	21
	23b	Discuss any limitations of the evidence included in the review.	22–23

Section and topic	Item #	Checklist item	Location where item is reported (page)
	23c	Discuss any limitations of the review processes used.	22–23
	23d	Discuss implications of the results for practice, policy, and future research.	21–23
**OTHER INFORMATION**			
Registration and protocol	24a	Provide registration information for the review, including register name and registration number, or state that the review was not registered.	1, 3
	24b	Indicate where the review protocol can be accessed, or state that a protocol was not prepared.	3
	24c	Describe and explain any amendments to information provided at registration or in the protocol.	6
Support	25	Describe sources of financial or non-financial support for the review, and the role of the funders or sponsors in the review.	23–24
Competing interests	26	Declare any competing interests of review authors.	23
Availability of data, code and other materials	27	Report which of the following are publicly available and where they can be found: template data collection forms; data extracted from included studies; data used for all analyses; analytic code; any other materials used in the review.	24

Page numbers refer to the revised manuscript.

From: Page MJ, McKenzie JE, Bossuyt PM, et al. The PRISMA 2020 statement: an updated guideline for reporting systematic reviews. BMJ 2021;372:n71.

Supplementary Table S2. PRIOR checklist

Section and topic	#	Item	Location reported (page)
**TITLE**			
Title	1	Identify the report as an overview of reviews.	1
**ABSTRACT**			
Abstract	2	Provide a comprehensive and accurate summary of the purpose, methods, and results of the overview of reviews.	1
**INTRODUCTION**			
Rationale	3	Describe the rationale for conducting the overview of reviews in the context of existing knowledge.	2
Objectives	4	Provide an explicit statement of the objective(s) or question(s) addressed by the overview of reviews.	2
**METHODS**			
Eligibility criteria	5a	Specify the inclusion and exclusion criteria for the overview of reviews. If supplemental primary studies were included, this should be stated, with a rationale.	3, 5–6
	5b	Specify the definition of 'systematic review' as used in the inclusion criteria for the overview of reviews.	3
Information sources	6	Specify all databases, registers, websites, organizations, reference lists, and other sources searched or consulted to identify systematic reviews and supplemental primary studies (if included). Specify the date when each source was last searched or consulted.	2–3
Search strategy	7	Present the full search strategies for all databases, registers and websites, such that they could be reproduced. Describe any search filters and limits applied.	3
Selection process	8a	Describe the methods used to decide whether a systematic review or supplemental primary study (if included) met the inclusion criteria of the overview of reviews.	3
	8b	Describe how overlap in the populations, interventions, comparators, and/or outcomes of systematic reviews was identified and managed during study selection.	3, 5
Data collection process	9a	Describe the methods used to collect data from reports.	3–4
	9b	If applicable, describe the methods used to identify and manage primary study overlap at the level of the comparison and outcome during data collection. For each outcome, specify the method used to illustrate and/or quantify the degree of primary study overlap across systematic reviews.	5
	9c	If applicable, specify the methods used to manage discrepant data across systematic reviews during data collection.	3, 6
Data items	10	List and define all variables and outcomes for which data were sought. Describe any assumptions made and/or measures taken to identify and clarify missing or unclear information.	3–6
Risk of bias assessment	11a	Describe the methods used to assess risk of bias or methodological quality of the included systematic reviews.	4–5

Section and topic	#	Item	Location reported (page)
	11b	Describe the methods used to collect data on (from the systematic reviews) and/or assess the risk of bias of the primary studies included in the systematic reviews. Provide a justification for instances where flawed, incomplete, or missing assessments are identified but not re-assessed.	5, 23
	11c	Describe the methods used to assess the risk of bias of supplemental primary studies (if included).	Not applicable
Synthesis methods	12a	Describe the methods used to summarize or synthesize results and provide a rationale for the choice(s).	5
	12b	Describe any methods used to explore possible causes of heterogeneity among results.	5–6
	12c	Describe any sensitivity analyses conducted to assess the robustness of the synthesized results.	Not applicable
Reporting bias assessment	13	Describe the methods used to collect data on (from the systematic reviews) and/or assess the risk of bias due to missing results in a summary or synthesis (arising from reporting biases at the levels of the systematic reviews, primary studies, and supplemental primary studies, if included).	3–4
Certainty assessment	14	Describe the methods used to collect data on (from the systematic reviews) and/or assess certainty (or confidence) in the body of evidence for an outcome.	3
**RESULTS**			
Systematic review and supplemental primary study selection	15a	Describe the results of the search and selection process, including the number of records screened, assessed for eligibility, and included in the overview of reviews, ideally with a flow diagram.	6–7
	15b	Provide a list of studies that might appear to meet the inclusion criteria, but were excluded, with the main reason for exclusion.	6
Characteristics of systematic reviews and supplemental primary studies	16	Cite each included systematic review and supplemental primary study (if included) and present its characteristics.	7–10
Primary study overlap	17	Describe the extent of primary study overlap across the included systematic reviews.	14–16
Risk of bias in systematic reviews, primary studies, and supplemental primary studies	18a	Present assessments of risk of bias or methodological quality for each included systematic review.	11–14
	18b	Present assessments (collected from systematic reviews or assessed anew) of the risk of bias of the primary studies included in the systematic reviews.	14
	18c	Present assessments of the risk of bias of supplemental primary studies (if included).	Not applicable
Summary or synthesis of results	19a	For all outcomes, summarize the evidence from the systematic reviews and supplemental primary studies (if included). If meta-analyses were done, present for each the summary estimate and its precision and measures of statistical heterogeneity. If comparing groups, describe the direction of the effect.	16–19
	19b	If meta-analyses were done, present results of all investigations of possible causes of heterogeneity.	Not applicable
	19c	If meta-analyses were done, present results of all sensitivity analyses conducted to assess the robustness of synthesized results.	Not applicable

Section and topic	#	Item	Location reported (page)
Reporting biases	20	Present assessments (collected from systematic reviews and/or assessed anew) of the risk of bias due to missing primary studies, analyses, or results in a summary or synthesis (arising from reporting biases at the levels of the systematic reviews, primary studies, and supplemental primary studies, if included) for each summary or synthesis assessed.	11
Certainty of evidence	21	Present assessments (collected or assessed anew) of certainty (or confidence) in the body of evidence for each outcome.	11, 18–19
**DISCUSSION**			
Discussion	22a	Summarize the main findings, including any discrepancies in findings across the included systematic reviews and supplemental primary studies (if included).	20–21
	22b	Provide a general interpretation of the results in the context of other evidence.	21
	22c	Discuss any limitations of the evidence from systematic reviews, their primary studies, and supplemental primary studies (if included) included in the overview of reviews. Discuss any limitations of the overview of reviews methods used.	22–23
	22d	Discuss implications for practice, policy, and future research (both systematic reviews and primary research). Consider the relevance of the findings to the end users of the overview of reviews, e.g., healthcare providers, policymakers, patients, among others.	21–23
**OTHER INFORMATION**			
Registration and protocol	23a	Provide registration information for the overview of reviews, including register name and registration number, or state that the overview of reviews was not registered.	1, 3
	23b	Indicate where the overview of reviews protocol can be accessed, or state that a protocol was not prepared.	3
	23c	Describe and explain any amendments to information provided at registration or in the protocol. Indicate the stage of the overview of reviews at which amendments were made.	6
Support	24	Describe sources of financial or non-financial support for the overview of reviews, and the role of the funders or sponsors in the overview of reviews.	23–24
Competing interests	25	Declare any competing interests of the overview of reviews' authors.	23
Author information	26a	Provide contact information for the corresponding author.	1
	26b	Describe the contributions of individual authors and identify the guarantor of the overview of reviews.	23
Availability of data and other materials	27	Report which of the following are available, where they can be found, and under which conditions they may be accessed: template data collection forms; data collected from included systematic reviews and supplemental primary studies; analytic code; any other materials used in the overview of reviews.	24

Page numbers refer to the revised manuscript.

From: Gates M, Gates A, Pieper D, et al. Reporting guideline for overviews of reviews of healthcare interventions: development of the PRIOR statement. BMJ 2022;378:e070849.

Supplementary Table S3. Search strategy

Database	Search number	Query	Result	Date
PubMed	1	("Pancreas/injuries"[Mesh] OR (("Pancreas"[Mesh] OR "Pancreatic Ducts"[Mesh] OR pancreas[tiab] OR pancreatic[tiab] OR pancreat*[tiab]) AND ("Wounds and Injuries"[Mesh] OR "Abdominal Injuries"[Mesh] OR trauma*[tiab] OR injur*[tiab] OR wound*[tiab] OR transect*[tiab] OR lacerat*[tiab] OR rupture*[tiab] OR blunt[tiab] OR penetrat*[tiab] OR gunshot[tiab] OR stab*[tiab])))	37,960	2026-09-03
	2	((("Meta-Analysis as Topic"[Mesh] OR meta-analys*[TW] OR metaanalys*[TW] OR "Meta-Analysis"[PT] OR Systematic-review*[TW] OR Systematic-overview*[TW] OR Systematic-literatur*[TW] OR "Systematic Reviews as Topic"[Mesh] OR "cochrane"[TIAB] OR "embase"[TIAB] OR "MEDLINE"[TIAB] OR "psychlit"[TIAB] OR "psyclit"[TIAB] OR "psychinfo"[TIAB] OR "psycinfo"[TIAB] OR "cinahl"[TIAB] OR "science citation index"[TIAB] OR "Scopus"[TIAB] OR "Web of science*"[TW] OR "hand-search*"[TW] OR "relevant journals"[TW] OR "manual search*"[TW] OR (("selection criteria"[TW] OR "data extraction"[TW]) AND "Review"[PT:NoExp])) NOT ("Comment"[PT] OR "Letter"[PT] OR "Editorial"[PT] OR ("Animals"[Mesh] NOT ("Humans"[Mesh] AND "Animals"[Mesh])))) OR systematic[sb] OR "Systematic Review"[PT])	810,418	2026-09-03
		#1 AND #2	685	2026-09-03
EMBASE	1	('pancreas injury'/exp OR (('pancreas'/exp OR 'pancreas duct'/exp OR pancrea*:ti,ab,kw) AND ('trauma'/exp OR 'abdominal injury'/exp OR trauma*:ti,ab,kw OR injur*:ti,ab,kw OR wound*:ti,ab,kw OR transect*:ti,ab,kw OR lacerat*:ti,ab,kw OR rupture*:ti,ab,kw OR blunt:ti,ab,kw OR penetrat*:ti,ab,kw OR gunshot:ti,ab,kw OR stab*:ti,ab,kw)))	83,564	2026-09-03
	2	(([systematic review]/lim OR [meta analysis]/lim OR 'meta analysis'/exp OR 'meta analysis (topic)'/exp OR 'systematic review'/exp OR 'systematic review (topic)'/exp OR (meta-analys* OR metaanalys* OR Systematic-review* OR Systematic-overview* OR Systematic-literatur*):ab,ti,kw) AND ([article]/lim OR [article in press]/lim OR [review]/lim))	760,437	2026-09-03
		#1 AND #2	1,673	2026-09-03
Cochrane	1	pancrea*:ti,ab,kw OR [mh Pancreas]	23,810	2026-09-03
	2	trauma*:ti,ab,kw OR injur*:ti,ab,kw OR wound*:ti,ab,kw OR [mh "Wounds and Injuries"]	170,930	2026-09-03
		#1 AND #2	29	2026-09-03

Supplementary Table S4. Systematic reviews excluded after full-text assessment, with reasons (n = 16)

No.	First author (year)	Title	Study design	Reason for exclusion
1	Benifla (2003)	Acute pancreatitis in childhood: analysis of literature data	Literature review (MEDLINE, 1965 onwards)	Ineligible population or outcome
2	Guesmi (2009)	Pancreatic pseudocysts located in the liver: a systematic review of the literature	Case report and systematic review of case reports	Ineligible population or outcome
3	Haugaard (2014)	Non-operative versus operative treatment for blunt pancreatic trauma in children	Cochrane systematic review restricted to randomised trials	No included studies (empty review)
4	Gurusamy (2016)	Management strategies for pancreatic pseudocysts	Cochrane systematic review	Ineligible population or outcome
5	Zhao (2016)	Endoscopic versus surgical treatment for pancreatic pseudocyst	Systematic review and meta-analysis	Ineligible population or outcome
6	Søreide (2018)	Clinical update on management of pancreatic trauma	Clinical update based on a literature search	Ineligible study design
7	Bukowski (2020)	Endoscopic stenting in grade III pancreatic injury according to the American Association for the Surgery of Trauma: a systematic review of case reports and case series	Systematic review of case reports and case series	Review restricted to case reports or case series
8	de Carvalho (2020)	Pancreaticodudonectomy in trauma: one or two stages?	Systematic review	Ineligible population or outcome
9	Jurkovich (2020)	Pancreatic trauma	Invited lecture (narrative review)	Ineligible study design
10	Catellani (2023)	Laparoscopic management of blunt pancreatic trauma in adults and pediatric patients: a systematic review	Case report and systematic review of reported cases	Review restricted to case reports or case series
11	Ehrsam (2024)	Foreign glass bodies in pleura and pancreas: systematic review for entry hypotheses and treatment options in an unresolved case	Case report and systematic review of case reports	Review restricted to case reports or case series
12	Kim (2024)	Pancreaticoduodenectomy for trauma in an adolescent female with complex pancreatic and duodenal injuries: case report and review of the literature	Case report and literature review	Ineligible study design
13	Sukhotnik (2024)	Management of blunt pancreatic trauma in children: a persistent controversy – case report and comprehensive literature review	Case report and literature review	Ineligible study design
14	Karpuzcu (2025)	The clinical efficacy and safety of ERCP in traumatic pancreatic injuries: a systematic review and meta-analysis	Systematic review and meta-analysis	Ineligible intervention or comparison
15	van Zon (2025)	New-onset diabetes mellitus following pancreatic injury: a systematic review	Systematic review	Ineligible population or outcome
16	Jaiswal (2026)	Robotic surgery in acute care: a systematic review of its role in visceral trauma and emergency general surgery	Systematic review	Ineligible population or outcome

Abbreviations: AAST, American Association for the Surgery of Trauma; ERCP, endoscopic retrograde cholangiopancreatography.

Supplementary Table S5. Operational definitions of OM and NOM in the included reviews

Review	OM definition	NOM definition	ERCP/stent	Percutaneous drainage	Surgery for other organs	Operative drainage	Estimates affected
Ho 2017	Not defined	Not defined; failure of NOM kept in the NOM arm	NOM (by example)	NR	NR	OM (non-resectional)	Adult mortality RR 0.67; fistula RR 0.49
Koh 2017	Immediate surgery after diagnosis of pancreatic injury	Observation, endoscopic or IR procedures, surgery for other organ injuries	NOM	NOM	NOM	OM	Pseudocyst 18% vs 4%; NOM success; LOS
Soon 2019	Laparotomy and any pancreas-related procedure	Supportive care to radiologically or endoscopically guided drainage	NOM	NOM	Not stated	NR	Mortality 28.8% vs 0.5%; LOS; reintervention
Kopljar 2021	Immediate surgery after diagnosis of pancreatic injury	Observation, medical, endoscopic or IR procedures, surgery for other organ systems	NOM	NOM	NOM	OM	Mortality OR 0.96 (pancreas-related deaths only); pseudocyst; fistula; LOS; TPN
Barrientos 2025	Not defined	Medical therapy, ERCP, stent, percutaneous or surgical drainage, supportive care	NOM	NOM	NR	NOM	All pooled estimates (mortality, complications, pseudocyst, fistula, reintervention)
Hus 2025	Not defined	Conservative, ERCP, percutaneous drainage	NOM	NOM	NR	OM	Narrative comparisons; one penetrating resection vs non-resection study presented as NOM vs OM

Abbreviations: IR, interventional radiology; LOS, length of stay; NOM, non-operative management; NR, not reported; OM, operative management; OR, odds ratio; RR, risk ratio; TPN, total parenteral nutrition.

Supplementary Table S6. Primary studies included in the systematic reviews (n = 95)

No.	First author	Year	Title	Journal	Vol	Issue	Pages	Included in systematic review
1	Abbo	2013	Conservative management of blunt pancreatic trauma in children: a single center experience	Eur J Pediatr Surg	23	6	470–473	Koh (2017); Kopljar (2021)
2	Addison	2016	Outcomes following operative vs. non-operative management of blunt traumatic pancreatic injuries: a retrospective multi-institutional study	Burns Trauma	4		39	Soon (2019)
3	Ahmad	2008	Pancreatic injuries in the United Arab Emirates	Scand J Surg	97	3	243–247	Ho (2017)
4	Al-Ahmadi	2008	Outcomes after pancreatic trauma: experience at a single institution	Can J Surg	51	2	118–124	Ho (2017)
5	Amirata	1994	Octreotide acetate decreases pancreatic complications after pancreatic trauma	Am J Surg	168	4	345–347	Ho (2017)
6	Arkovitz	1997	Pancreatic trauma in children: mechanisms of injury	J Trauma	42	1	49–53	Koh (2017); Kopljar (2021)
7	Bass	1991	Pancreatic injuries in children	S Afr J Surg	29	2	39–40	Kopljar (2021)
8	Beger	2019	Pancreatic injury due to blunt abdominal trauma in children	Med Sci (Turkey)	8	1	225–228	Kopljar (2021)
9	Ben Hassine	2010	[Management of blunt duodenal and pancreatic injuries in children (about a series of 8 cases)]	Tunis Med	88	8	586–592	Kopljar (2021); Barrientos (2025)
10	Beres	2013	Non-operative management of high-grade pancreatic trauma: is it worth the wait?	J Pediatr Surg	48	5	1060–1064	Koh (2017); Kopljar (2021); Barrientos (2025); Hus (2025)
11	Biffl	2021	A multicenter trial of current trends in the diagnosis and management of high-grade pancreatic injuries	J Trauma Acute Care Surg	90	5	776–786	Hus (2025)
12	Biffl	2022	A comparison of management and outcomes following blunt versus penetrating pancreatic trauma: a secondary analysis from the Western Trauma Association Multicenter Trials Group on Pancreatic Injuries	J Trauma Acute Care Surg	93	5	620–626	Hus (2025)
13	Borkon	2011	Operative intervention for complete pancreatic transection in children sustaining blunt abdominal trauma: revisiting an organ salvage technique	Am Surg	77	5	612–620	Koh (2017); Kopljar (2021)
14	Canty	2001	Management of major pancreatic duct injuries in children	J Trauma	50	6	1001–1007	Koh (2017); Kopljar (2021)
15	Cigdem	2011	Nonoperative management of pancreatic injuries in pediatric patients	Surg Today	41	5	655–659	Koh (2017); Kopljar (2021); Barrientos (2025)
16	Cogbill	1991	Distal pancreatectomy for trauma: a multicenter experience	J Trauma	31	12	1600–1606	Ho (2017)
17	Cuenca	2012	Pediatric pancreatic trauma: trending toward nonoperative management?	Am Surg	78	11	1204–1210	Koh (2017); Kopljar (2021); Barrientos (2025)

No.	First author	Year	Title	Journal	Vol	Issue	Pages	Included in systematic review
18	Dai	2015	Abdominal injuries involving bicycle handlebars in 219 children: results of 8-year follow-up	Eur J Trauma Emerg Surg	41	5	551–555	Koh (2017); Kopljar (2021)
19	de Blaauw	2008	Pancreatic injury in children: good outcome of nonoperative treatment	J Pediatr Surg	43	9	1640–1643	Kopljar (2021); Barrientos (2025)
20	Degiannis	1995	Distal pancreatectomy for gunshot injuries of the distal pancreas	Br J Surg	82	9	1240–1242	Ho (2017)
21	Degiannis	1996	Gunshot injuries of the head of the pancreas: conservative approach	World J Surg	20	1	68–71	Ho (2017)
22	el-Boghdadly	2000	Pancreatic injury: an audit and a practical approach	Ann R Coll Surg Engl	82	4	258–262	Ho (2017)
23	Everson	2023	Paediatric pancreatic trauma in North Queensland: a 10-year retrospective review	BMC Pediatr	23	1	88	Barrientos (2025)
24	Fabbro	2001	Management of severe blunt pancreatic trauma in children. Personal experience	Pediatr Med Chir	23	3–4	179–182	Kopljar (2021)
25	Fabian	1990	Superiority of closed suction drainage for pancreatic trauma. A randomized, prospective study	Ann Surg	211	6	724–728	Ho (2017)
26	Firstenberg	1999	Selective management of pediatric pancreatic injuries	J Pediatr Surg	34	7	1142–1147	Koh (2017); Kopljar (2021)
27	Garg	2017	Blunt trauma pancreas in children: is non-operative management appropriate for all grades?	Pediatr Gastroenterol Hepatol Nutr	20	4	252–258	Kopljar (2021); Barrientos (2025)
28	Garvey	2015	Role of ERCP in pediatric blunt abdominal trauma: a case series at a level one pediatric trauma center	J Pediatr Surg	50	2	335–338	Koh (2017)
29	Ghasoup	2013	Management of pancreatic injuries with blunt abdominal trauma in children: case series	J Trauma Treat	2		160	Kopljar (2021); Barrientos (2025)
30	Graham	2000	Pancreatic trauma in Scottish children	J R Coll Surg Edinb	45	4	223–226	Kopljar (2021)
31	Hamidian Jahromi	2013	Surgical versus nonsurgical management of traumatic major pancreatic duct transection: institutional experience and review of the literature	Pancreas	42	1	76–87	Ho (2017)
32	Heuer	2011	Pancreatic injury in 284 patients with severe abdominal trauma: outcome, course, and treatment algorithm	Langenbecks Arch Surg	396	7	1067–1076	Ho (2017)
33	Holland	1999	Traumatic pancreatitis: long-term review of initial non-operative management in children	J Paediatr Child Health	35	1	78–81	Kopljar (2021)
34	Houben	2007	Traumatic pancreatic duct injury in children: minimally invasive approach to management	J Pediatr Surg	42	4	629–635	Koh (2017)
35	Iqbal	2014	Operative vs nonoperative management for blunt pancreatic transection in children: multi-institutional outcomes	J Am Coll Surg	218	2	157–162	Koh (2017); Kopljar (2021); Barrientos (2025); Hus (2025)

No.	First author	Year	Title	Journal	Vol	Issue	Pages	Included in systematic review
36	Ivatury	1990	Penetrating pancreatic injuries. Analysis of 103 consecutive cases	Am Surg	56	2	90–95	Ho (2017)
37	Ivatury	1993	The spleen at risk after penetrating trauma	J Trauma	35	3	409–414	Ho (2017)
38	Jacombs	2004	Pancreatic trauma in children	J Pediatr Surg	39	1	96–99	Koh (2017)
39	Jobst	1999	Management of pancreatic injury in pediatric blunt abdominal trauma	J Pediatr Surg	34	5	818–823	Kopljar (2021)
40	Juric	2009	Management of blunt pancreatic trauma in children	Surg Today	39	2	115–119	Koh (2017); Kopljar (2021)
41	Kaman	2012	Current management of pancreatic trauma	Trop Gastroenterol	33	3	200–206	Ho (2017)
42	Katsura	2024	Recent changes in the management of high-grade blunt pancreatic injury in children: a nationwide trend analysis	J Am Coll Surg	238	6	1106–1114	Hus (2025)
43	Katz	2018	Surgical outcomes of pancreaticoduodenal injuries in children	Pediatr Surg Int	34	6	641–645	Kopljar (2021); Barrientos (2025)
44	Keil	2016	What is the role of endoscopic retrograde cholangiopancreatography in assessing traumatic rupture of the pancreatic in children?	Scand J Gastroenterol	51	2	218–224	Kopljar (2021); Barrientos (2025)
45	Keller	1997	Conservative management of pancreatic trauma in children	J Trauma	42	6	1097–1100	Kopljar (2021)
46	Kim	2001	The role of endoscopic retrograde pancreatography in the treatment of traumatic pancreatic duct injury	Gastrointest Endosc	54	1	49–55	Ho (2017)
47	Klin	2011	Blunt pancreatic trauma in children	Surg Today	41	7	946–954	Koh (2017); Kopljar (2021); Barrientos (2025)
48	Koganti	2016	Predictors of successful non-operative management of grade III & IV blunt pancreatic trauma	Ann Med Surg	10		103–109	Soon (2019); Hus (2025)
49	Kong	2013	Grading-therapeutic strategy for pancreatic injury after blunt abdominal trauma: therapy based on the condition of pancreatic duct and report of 95 cases	Hepatogastroenterology	60	126	1497–1503	Ho (2017)
50	Kong	2014	Endoscopic management for pancreatic injuries due to blunt abdominal trauma decreases failure of nonoperative management and incidence of pancreatic-related complications	Injury	45	1	134–140	Soon (2019)
51	Kouchi	1999	Non-operative management of blunt pancreatic injury in childhood	J Pediatr Surg	34	11	1736–1739	Kopljar (2021)
52	Krige	2011	Pancreatic injuries after blunt abdominal trauma: an analysis of 110 patients treated at a level 1 trauma centre	S Afr J Surg	49	2	58, 60, 62–64	Ho (2017)
53	Krige	2014	An analysis of predictors of morbidity after stab wounds of the pancreas in 78 consecutive injuries	Ann R Coll Surg Engl	96	6	427–433	Hus (2025)

No.	First author	Year	Title	Journal	Vol	Issue	Pages	Included in systematic review
54	Krige	2015	Prognostic factors, morbidity and mortality in pancreatic trauma: a critical appraisal of 432 consecutive patients treated at a Level 1 Trauma Centre	Injury	46	5	830–836	Hus (2025)
55	Kuza	2020	Pancreatic injuries in abdominal trauma in US adults: analysis of the National Trauma Data Bank on management, outcomes, and predictors of mortality	Scand J Surg	109	3	193–204	Hus (2025)
56	Lee	2012	Management of blunt pancreatic injury by applying the principles of damage control surgery: experience at a single institution	Hepatogastroenterology	59	118	1970–1975	Ho (2017)
57	Lee	2012	Outcomes of hemodynamically stable patients with pancreatic injury after blunt abdominal trauma	Pancreatology	12	6	487–492	Ho (2017)
58	Lewis	1991	Conservative surgery for trauma to the pancreatic head: is it safe?	Injury	22	5	372–374	Ho (2017)
59	Lin	2004	Management of blunt major pancreatic injury	J Trauma	56	4	774–778	Ho (2017)
60	Lin	2006	Long-term results of endoscopic stent in the management of blunt major pancreatic duct injury	Surg Endosc	20	10	1551–1555	Ho (2017)
61	Lin	2022	Resection versus drainage in the management of patients with AAST-OIS grade IV blunt pancreatic injury: a single trauma centre experience	Injury	53	1	129–136	Hus (2025)
62	Loungnarath	2001	Blunt pancreatic injuries in children	Ann Chir	126	10	992–995	Kopljar (2021)
63	Lu	2023	Management and outcome of blunt pancreatic trauma: a retrospective cohort study	World J Surg	47	9	2135–2144	Hus (2025)
64	Mattix	2007	Pediatric pancreatic trauma: predictors of nonoperative management failure and associated outcomes	J Pediatr Surg	42	2	340–344	Koh (2017); Kopljar (2021); Barrientos (2025); Hus (2025)
65	Meier	2001	Early operation in children with pancreas transection	J Pediatr Surg	36	2	341–344	Kopljar (2021)
66	Mohseni	2018	Outcomes after resection versus non-resection management of penetrating grade III and IV pancreatic injury: a trauma quality improvement (TQIP) databank analysis	Injury	49	1	27–32	Hus (2025)
67	Mora	2016	Operative vs nonoperative management of pediatric blunt pancreatic trauma: evaluation of the National Trauma Data Bank	J Am Coll Surg	222	6	977–982	Kopljar (2021); Barrientos (2025); Hus (2025)
68	Nadler	1999	Management of blunt pancreatic injury in children	J Trauma	47	6	1098–1103	Kopljar (2021)
69	Nance	1971	Pancreaticoduodenectomy following abdominal trauma	J Trauma	11	7	577–585	Ho (2017)
70	Nwariaku	1995	Is octreotide beneficial following pancreatic injury?	Am J Surg	170	6	582–585	Ho (2017)

No.	First author	Year	Title	Journal	Vol	Issue	Pages	Included in systematic review
71	Olah	2003	Pancreatic transection from blunt abdominal trauma: early versus delayed diagnosis and surgical management	Dig Surg	20	5	408–414	Ho (2017)
72	Pachter	1989	Traumatic injuries to the pancreas: the role of distal pancreatectomy with splenic preservation	J Trauma	29	10	1352–1355	Ho (2017)
73	Pata	2009	Extension of nonoperative management of blunt pancreatic trauma to include grade III injuries: a safety analysis	World J Surg	33	8	1611–1617	Ho (2017)
74	Paul	2011	The management of pancreatic injuries in children: operate or observe	J Pediatr Surg	46	6	1140–1143	Koh (2017); Kopljar (2021); Barrientos (2025)
75	Rauh	2024	Contemporary management and outcomes of blunt traumatic American Association for the Surgery of Trauma organ injury scale grades III and IV pancreatic injuries in children: a Trauma Quality Improvement Program analysis	J Trauma Acute Care Surg	97	3	365–370	Hus (2025)
76	Rickard	2005	Pancreatic and duodenal injuries: keep it simple	ANZ J Surg	75	7	581–586	Ho (2017)
77	Robey	1982	Blunt transection of the pancreas treated by distal pancreatectomy, splenic salvage and hyperalimentation. Four cases and review of the literature	Ann Surg	196	6	695–699	Ho (2017)
78	Ruszinko	2005	Pancreatic injury from blunt abdominal trauma in childhood	Acta Chir Belg	105	3	283–286	Kopljar (2021)
79	Salam	1972	Pancreatoduodenectomy for trauma: clinical and metabolic studies	Ann Surg	175	5	663–672	Ho (2017)
80	Shaked	2003	Management of blunt pancreatic injuries in children	Eur J Trauma	29	3	151–155	Kopljar (2021)
81	Shilyansky	1998	Non-operative management of pancreatic injuries in children	J Pediatr Surg	33	2	343–349	Kopljar (2021)
82	Snajdauf	2007	Surgical management of major pancreatic injury in children	Eur J Pediatr Surg	17	5	317–321	Koh (2017); Kopljar (2021)
83	Sriussadaporn	1994	Management of pancreatic injuries	J Med Assoc Thai	77	11	580–587	Ho (2017)
84	Stringer	2005	Pancreatic trauma in children	Br J Surg	92	4	467–470	Koh (2017); Kopljar (2021)
85	Sukul	1992	Management of pancreatic injuries	Hepatogastroenterology	39	5	447–450	Ho (2017)
86	Sutherland	2010	Pancreatic trauma in children	Pediatr Surg Int	26	12	1201–1206	Koh (2017)
87	Takishima	1996	Characteristics of pancreatic injury in children: a comparison with such injury in adults	J Pediatr Surg	31	7	896–900	Kopljar (2021)
88	Teh	2007	Diagnosis and management of blunt pancreatic ductal injury in the era of high-resolution computed axial tomography	Am J Surg	193	5	641–643	Ho (2017)
89	Timberlake	1997	Blunt pancreatic trauma: experience at a rural referral center	Am Surg	63	3	282–286	Ho (2017)
90	Vane	2009	Initial resection of potentially viable tissue is not optimal treatment for grades II–IV pancreatic injuries	World J Surg	33	2	221–227	Koh (2017); Kopljar (2021); Barrientos (2025)

No.	First author	Year	Title	Journal	Vol	Issue	Pages	Included in systematic review
91	Wales	2001	Long-term outcome after nonoperative management of complete traumatic pancreatic transection in children	J Pediatr Surg	36	5	823–827	Koh (2017); Kopljar (2021)
92	Wood	2010	Operative vs nonoperative management of blunt pancreatic trauma in children	J Pediatr Surg	45	2	401–406	Koh (2017); Kopljar (2021); Barrientos (2025); Hus (2025)
93	Yadav	2007	Spleen-preserving distal pancreatectomy for pancreatic trauma: a series of six cases	JOP	8	4	422–428	Ho (2017)
94	Yamana	2012	Pancreatic injury in children: review of 7 cases and the pertinent literature	Hepatogastroenterology	59	114	574–577	Koh (2017); Kopljar (2021)
95	Young	1998	Pancreatic injuries resulting from penetrating trauma: a multi-institution review	Am Surg	64	9	838–843	Ho (2017)

Abbreviations: ERCP, endoscopic retrograde cholangiopancreatography.

Supplementary Table S7. Representative review for each pediatric outcome

Outcome	Representative review (basis)	Concordance check
Mortality	Kopljar 2021 (32 studies; pooled)	Barrientos 2025 (6 studies); Hus 2025
Pancreatic fistula	Kopljar 2021 (7 studies; pooled)	Barrientos 2025 (6 studies)
Pseudocyst, overall and grade ≥ III	Kopljar 2021 (10 studies in MA; pooled)	Barrientos 2025 (8 studies); Koh 2017
NOM success	Kopljar 2021 (42 studies)	Barrientos 2025; Koh 2017
LOS, TPN, readmission	Kopljar 2021 (only pooled source)	Koh 2017 (descriptive)
Reintervention	Barrientos 2025 (15 studies; only pooled source)	–
Overall complications	Barrientos 2025 (17 studies; only pooled source)	Koh 2017 (descriptive)
ICU admission; hemorrhage; infection; pancreatitis	Barrientos 2025 (only pooled source)	–
Grade-stratified NOM success; pseudocyst management	Koh 2017 (only source)	–

Abbreviations: ICU, intensive care unit; LOS, length of stay; MA, meta-analysis; NOM, non-operative management; TPN, total parenteral nutrition.
